# Reduced m6A modification predicts malignant phenotypes and augmented Wnt/PI3K‐Akt signaling in gastric cancer

**DOI:** 10.1002/cam4.2360

**Published:** 2019-06-26

**Authors:** Cheng Zhang, Mengqi Zhang, Sai Ge, Wenwen Huang, Xiaoting Lin, Jing Gao, Jifang Gong, Lin Shen

**Affiliations:** ^1^ Department of Gastrointestinal Oncology, Key Laboratory of Carcinogenesis and Translational Research (Ministry of Education/Beijing) Peking University Cancer Hospital & Institute Beijing China

**Keywords:** gastric cancer, m6A, tumor suppressor, Wnt/PI3K‐Akt signaling

## Abstract

**Background:**

As the most abundant epigenetic modification on mRNAs and long non‐coding RNAs, N6‐methyladenosine (m6A) modification extensively exists in mammalian cells. Controlled by writers (methyltransferases), readers (signal transducers), and erasers (demethylases), m6A influences mRNA structure, maturation, and stability, thus negatively regulating protein expression in a post‐translational manner. Nevertheless, current understanding of m6A's roles in tumorigenesis, especially in gastric cancer (GC) remains to be unveiled. In this study, we assessed m6A's clinicopathological relevance to GC and explored the underlying mechanisms.

**Methods:**

By referring to a proteomics‐based GC cohort we previously generated and the TCGA‐GC cohort, we merged expressions of canonical m6A writers (METTL3/METTL14), readers (YTHDF1/YTHDF2/YTHDF3), and erasers (ALKBH5/FTO), respectively, as W, R, and E signatures to represent m6A modification. We stratified patients according to these signatures to decipher m6A's associations with crucial mutations, prognosis, and clinical indexes. m6A's biological functions in GC were predicted by gene set enrichment analysis (GSEA) and validated by in vitro experiments.

**Results:**

We discovered that W and R were potential tumor suppressive signatures, while E was a potential oncogenic signature in GC. According to W/R/E stratifications, patients with low m6A‐indications were accompanied with higher mutations of specific genes (*CDH1*, *AR*, *GLI3*, *SETBP1*, *RHOA*, *MUC6*, and *TP53*) and also demonstrated adverse clinical outcomes. GSEA suggested that reduced m6A was correlated with oncogenic signaling and phenotypes. Through in vitro experiments, we proved that m6A suppression (represented by METTL14 knockdown) promoted GC cell proliferation and invasiveness through activating Wnt and PI3K‐Akt signaling, while m6A elevation (represented by FTO knockdown) reversed these phenotypical and molecular changes. m6A may also be involved in interferon signaling and immune responses of GC.

**Conclusions:**

Our work demonstrated that low‐m6A signatures predicted adverse clinicopathological features of GC, while the reduction of RNA m6A methylation activated oncogenic Wnt/PI3K‐Akt signaling and promoted malignant phenotypes of GC cells.

## 1 INTRODUCTION

The activity of RNA is tightly controlled by multiple types of post‐transcriptional regulations, such as chemical modifications and structural alterations, which influence RNA stability and degradation, subsequently induce protein translation changes and modulate signaling pathways. Over 100 post‐transcriptional modifications on RNA have been identified, in which N6‐methyladenosine (m6A) is the most predominant modification of mRNA and long noncoding RNA observed in high eukaryotic cells.^1^ In both eukaryotes and prokaryotes, m6A also widely exists in other types of RNAs, including ribosomal RNAs, small nuclear RNAs, and transfer RNAs.^2^

With the understanding to RNA methylation deepens, a series of regulators have been identified to be involved in regulating mammalian m6A. m6A modification on RNA was imprinted by methyltransferases, preferentially recognized and conveyed by reader proteins, and erased by RNA demethylases. Consequently, these three categories of regulators dynamically controlling m6A are defined as writers, readers and erasers. Controlled by these three types of regulators, m6A methylation epigenetically mediates expression of vast genes, thus playing multiple roles in modulating biological processes. Acquisition of m6A reduces transcript stability and mediates target mRNA decay, suggesting m6A modification as a negative regulator of mRNA translation. Conversely, loss of m6A enhanced the abundance and lifespan of transcripts, as well as the overall expression of proteins. m6A also alters RNA structure, facilitates the binding of protein regulators, influences mRNA maturation and modulates gene expression.^3^

A variety of proteins have been proved to be involved in m6A regulation, in which METTL3, METTL14 were identified as writers, YTHDF1, YTHDF2, YTHDF3 as readers, and FTO, ALKBH5 as erasers.^4^ As two classical S‐adenosyl methionine‐binding subunit associated with mRNA methylation, METTL3 and METTL14 form a heterodimer complex that mediates m6A deposition on mammalian mRNAs.^5^, ^6^ METTL3 knockdown simultaneously elicited a reduction in m6A and apoptosis of HeLa cells,^2^ while knockout of its interactive homologue METTL14 in mouse embryonic stem cells displayed a resistance to maturation.^7^, ^8^ Among YTH domain‐containing proteins, YTHDF1/YTHDF2/YTHDF3 were identified as m6A cytosolic readers, who selectively bind to the m6A on the transcripts of its target via their C‐terminal YTH domain.^4^, ^9^ It was shown that YTHDF2 promoted tumor suppressive SCOCS2 degradation in hepatocellular carcinoma.^10^ For erasers, FTO removes the m6A residues around the splice sites and modulates the alternative splicing of its target transcripts.^11^ FTO‐deficient mice exhibited early mortality and reduced body mass, while ALKBH5 knockout mice displayed impaired male fertility.^12^, ^13^, ^14^ m6A modification is also found participating in cell fate determination and embryonic development.^6^

Recent studies also outlined m6A regulators' involvement in cancer. In breast cancer, Panneerdoss and colleagues pointed out that the interplay among writers, readers, and erasers determines the stability of a series of cell cycle, epithelial‐mesenchymal transition (EMT), and angiogenesis regulators.^1^ Hypoxia‐activated HIF‐1α/HIF‐2α upregulated ALKBH5, while depletion of ALKBH5 reduced metastasis via reducing cancer stem cells.^15^, ^16^ It was reported that IGF2BP1 sustains the expression of various SRF target genes and promotes liver cancer progression in an m6A‐dependent manner.^17^ 2%–9% copy number variation (CNV) of m6A regulator genes, including *METTL3*, *METTL14*, *YTHDF1*, *YTHDF2*, *ALKBH5*, and *FTO*, were observed in acute myeloid leukemia (AML), while patients carrying m6A‐related CNVs or mutations displayed poorer prognosis.^18^ Increased m6A enhances the expression of PTEN/c‑Myc/BCL2, modulates myeloid differentiation, haematopoietic stem/progenitor cell specification, while FTO‐mediated m6A inhibition promotes leukocyte transformation and leukemogenesis.^19^, ^20^, ^21^, ^22^ Upregulation of ALKBH5 or knockdown of METTL3/METTL14 were also reported to induce glioblastoma tumorigenesis by promoting FOXM1 expression.^23^, ^24^

However, the clinicopathological effects and relevant mechanisms of RNA m6A modification, especially in gastric cancer (GC), remain largely unrevealed. In this study, we assessed the clinical correlation of m6A modification in GC patient cohorts, and identified the pathways and phenotypes regulated by m6A modification. Our work expanded current understanding to m6A‐related signaling, and provided novel insights for the realm of GC research.

## 2 MATERIALS AND METHODS

### 2.1 Origin of patient specimens and datasets

Tissue specimens from 78 diffuse GC patients (testified by both mass‐spectrum (MS) based profiling and exome sequencing) were collected by Department of gastrointestinal oncology and Department of Pathology, Peking University Cancer Hospital & Institute. Experiments using patient specimens were approved by the Institutional Ethics Committee, Peking University Cancer Hospital & Institute. Written informed consents were obtained from all specimen providers. Proteomic profiling as well as exome sequencing for the samples were previously performed and described.^25^ Additionally, we were free to download and analyze the GC TCGA dataset with R 3.0.2 software (www.r-project.org). The data that support the findings of this study are openly available in TCGA (https://portal.gdc.cancer.gov/) and reference number (25).

### 2.2 Stratification and definition of m6A signatures

In both MS and TCGA datasets, the geometric average between METTL3 and METTL14's expressions was calculated and used as the writer signature (namely, W), similarly, YTHDF1, YTHDF2, YTHDF3 were combined as the reader signature (namely, R), while ALKBH5 and FTO were combined as the eraser signature (namely, E). Patients were classified into “low” or “high” groups according to expressions of the seven m6A regulators (METTL3, METTL14, YTHDF1, YTHDF2, YTHDF3, ALKBH5, FTO) or the three signatures (W, R, E) in a median‐based criterion. Following the same criterion, W and E were concomitantly stratified into writer‐low‐eraser‐low (W^L^E^L^), writer‐low‐eraser‐high (W^L^E^H^), writer‐high‐eraser‐low (W^H^E^L^), writer‐high‐eraser‐high (W^H^E^H^) groups; R and E were concomitantly stratified into reader‐low‐eraser‐low (R^L^E^L^), reader‐low‐eraser‐high (R^L^E^H^), reader‐high‐eraser‐low (R^H^E^L^), reader‐high‐eraser‐high (R^H^E^H^) groups; W and R were concomitantly stratified into writer‐low‐reader‐low (W^L^R^L^), writer‐low‐reader‐high (W^L^R^H^), writer‐high‐reader‐low (W^H^R^L^), writer‐high‐reader‐high (W^H^R^H^) groups. When jointly considering the expressions of W, R, and E, we further stratified patients into six subgroups: writer and reader‐double‐low‐eraser‐low (WR^dL^E^L^), writer and reader‐double‐low‐eraser‐high (WR^dL^E^H^), writer and reader‐single‐high‐eraser‐low (WR^sH^E^L^), writer and reader‐single‐high‐eraser‐high (WR^sH^E^H^), writer and reader‐double‐high‐eraser‐low (WR^dH^E^L^), writer and reader‐double‐high‐eraser‐high (WR^dH^E^H^). Altogether, since writers/readers promoted while erasers counteracted m6A level and functions, W^L^, R^L^, E^H^, W^L^E^H^, R^L^E^H^, W^L^R^L^, and WR^dL^E^H^ stratifications were defined as low m6A‐indications, while W^H^, R^H^, E^L^, W^H^E^L^, R^H^E^L^, W^H^R^H^, and WR^dH^E^L^ as high m6A‐indications.

### 2.3 Statistics analysis and formatting

The seven m6A regulators or three signatures' mutual relationships were assessed with Pearson or Spearman correlation analysis. The diversity of mutations or expressions between stratified groups were compared with Student t‐test. Survival proportions (overall survival, OS) were compared with Kaplan‐Meier analysis paired with Log‐rank test. The best cut‐off value for Kaplan‐Meier analysis were calculated with ROC curves. Clinical indications were compared with Fisher's exact test or Chi‐square test. All statistics were performed with SPSS 21.0 software, and *P* < 0.05 was considered to be of statistical significance. Heatmaps and Venn diagrams were formatted with R 3.0.2 or Excel software. Other statistics were formatted with GraphPad Prism 5.1 software.

### 2.4 Gene set enrichment analysis

Categorical gene set enrichment analysis (GSEA) was performed with GSEA v2.0.13 software for stratifications based on W, R, E signatures or their double/triple stratifications. Official gene sets, including Hallmark (H), Oncogenic (C6) as well as three interferon‐related ones (type I interferon, interferon α/β and interferon γ), were downloaded from GSEA website (www.broadinstitute.org/gsea/) for enrichment. A permutation number of 1000 was adopted.

### 2.5 Cell lines, culturing and transfection

Gastric cancer cell lines HGC‐27 and MKN45 were purchased from ATCC (Manassas, VA). MGC803 was purchased from Shanghai Institutes for Biological Sciences (Shanghai, China). Cells were maintained in RPMI‐1640 medium (Invitrogen, Carlsbad, CA) supplemented with 10% fetal calf serum (Gibco BRL) and 1% penicillin plus streptomycin (HyClone, Logan, UT), and incubated in a humidified incubator (37℃, 5% CO2). Transfection of small interference RNAs (siRNAs) was mediated by Lipo 3000 transfection reagent (Thermo Fisher, USA). siRNAs were generated by Ribobio (Guangzhou, China). Interference sequences are: siR‐METTL14‐1, 5′‐GCATTGGTGCCGTGTTAAATA‐3′; siR‐METTL14‐2, 5′‐GGTTACAGAAGATGTGAAGAT‐3′; siR‐METTL14‐3, 5′‐GCTAATGTTGACATTGACTTA‐3′; siR‐YTHDF1‐1, 5′‐ACGGCAGAGTCGAAACAAA‐3′; siR‐YTHDF1‐2, 5′‐CTCCACCCATAAAGCATAA‐3′; siR‐YTHDF1‐3, 5′‐GCCGTCCATTGGATTTCCT‐3′; siR‐FTO‐1, 5′‐GGATGACTCTCATCTCGAA‐3′; siR‐FTO‐2, 5'‐GCTGAAATATCCTAAACTA‐3′; siR‐FTO‐3, 5′‐GTCACGAATTGCCCGAACA‐3′.

### 2.6 Cell proliferation assay

After transfected with siRNA sequences for 36 hours, cells were cultured in 96‐well plates (3 × 10^3^ per well) as triplicate wells and incubated under 37°C. After adherence (regarded as 0 hour time point), the proliferation rates of cells were measured with the CloneSelect Imager system (Molecular Devices, Sunnyvale, CA) at 24, 48, 72, and 96 hours time points, and formatted as growth curves with GraphPad Prism 5.1.

### 2.7 Migration and invasion assay

For migration assay, 150 μL re‐suspended cells (2 × 10^5^ per mL) were plated in the upper chamber of each transwell (Corning, New York, NY), then inserted into the bottom chamber containing 800 μL complete 1640 medium (10% FBS). After 48 hours culturing, the transwells were fixed in methanol and stained with 0.1% crystal violet solution. Cells remaining in the upper chamber were removed. For invasion assay, transwells pre‐coated with matrigel (Corning, New York, NY) were used instead of normal ones, while the other procedures were similar to those of migration assay. The polycarbonate membrane carrying penetrated cells was cut off, sealed with resin, and then observed under 200× microscope. Cells in captured images were counted with Image J, and measured with SPSS 21.0 software.

### 2.8 RNA purification, quantification of m6A methylation and quantitative RT‐PCR

RNA in cultured cells was extracted with total RNA purification kit (TR01, Genemark, China), and treated with DNase I to remove DNA contaminations. Purified RNA (200 ng per reaction) was assessed with m6A RNA methylation quantification kit (P‐9008, Epigentek, Farmingdale, NY). A negative and a positive control were provided along with the kit. Adjusted m6A levels were calculated following the provider's instructions.

After reverse transcription, quantitative PCR assay was performed using SYBR Green (Applied Biosystems, Foster City, CA) method. The setting for amplification was 95°C/30 s, followed by 40 cycles of 95°C/30 s, 59°C/20 s, and 72°C/30 s. Primers were generated by Sangon Biotechnology (Shanghai, China). Primer sequences were:

IFNA, 5′‐ATTTCTGCTCTGACAACCTC‐3′, 5′‐CTGAATGACTTGGAAGCCTG‐3′

IFNB, 5′‐ACTGCAACCTTTCGAAGCCT‐3′, 5′‐AGCCTCCCATTCAATTGCCA‐3′

IFNG, 5′‐TGCAGGTCATTCAGATGTAGCGGA‐3, 5′‐TGTCTTCCTTGATGGTCTCCACACTC‐3′

ISG15, 5′‐TTTGCCAGTACAGGAGCTTG‐3′, 5′‐TTCAGCTCTGACACCGACAT‐3′

GAPDH, 5′‐TGGTATCGTGGAAGGACTCA‐3′, 5′‐CCAGTAGAGGCAGGGATGAT‐3′

### 2.9 Western blot assay

Cells were directly lyzed in 1× boiling SDS‐PAGE loading buffer (1% SDS, 11% glycerol, 10% β‐mercaptoethanol, 0.1 mol/L Tris, pH 6.8). Protein bands were successively probed with primary antibodies and HRP‐conjugated secondary antibodies, then visualized using the Clarity Western ECL substrate (Bio‐Rad, Hercules, CA) and visualized with Amersham Imager 600 (GE Healthcare, Chicago, IL). Antibodies for E‐cadherin (ab40772), FTO (ab124892), and METTL14 (ab220030) were purchased from Abcam. Antibodies for Akt (#4691), pS473‐Akt (#4060), Axin (#2087), β‐catenin (#8480), GSK‐3β (#12456), pS9‐GSK‐3β (#5558), S6 (#2217), and pS235‐S6 (#4858) were from CST. Antibody for GAPDH (60004) and YTHDF1 (17479‐1‐AP) were from Proteintech.

### 2.10 ELISA assay

Cells were pre‐cultured in 24‐well plates and transfected with siRNAs. Transfected medium was refreshed 24 hours post transfection. Supernatant were collected 48 hours post transfection and testified with the Human IFN‐alpha ELISA Kit (41100‐1), Human IFN‐beta DuoSet ELISA Kit (DY814‐05) and Human IFN‐gamma DuoSet ELISA Kit (DY285B). All kits were acquired from R&D Systems.

## 3 RESULTS

### 3.1 Quantifying m6A regulators' expression profiles by mass spectrum profiling

An antibody capture‐based sequencing approach has been recently developed to identify m6A transcripts, yet m6A‐aimed omics study regarding patient‐derived cancer specimens remains rare.^26^ In order to investigate m6A's roles in GC, we extracted the expressions of m6A regulators from large‐sample GC cohort to represent the degree of m6A modification. According to previous reports, METTL3/METTL14, YTHDF1/YTHDF2/YTHDF3, and ALKBH5/FTO were certificated to be writers, readers, and erasers for m6A modification, respectively. These three classifications of m6A regulators were assessed across a mass spectrum‐based GC dataset (MS cohort) previously generated by our groups.^25^ In this dataset, members within the same classification were positively correlated on protein level (Figure S1A), validating the functions of group members were in consistency.

In order to better evaluate the impact of m6A modification in GC, we combined expressions of METTL3/METTL14 as the signature for writer (abbreviated as W), YTHDF1/YTHDF2/YTHDF3 as the signature for reader (abbreviated as R), and ALKBH5/FTO as the signature for eraser (abbreviated as E). In MS cohort, expression level of W displayed a positive correlation with R, while despite the statistics were insignificant due to limited samples, levels of W and R were both negatively correlated with that of E (Figure S1B), which comply with the fact that writers and readers were exclusive to erasers in mediating m6A modification and functions.

### 3.2 Expressional and mutational landscape of m6A regulators and signatures

**Figure 1:**
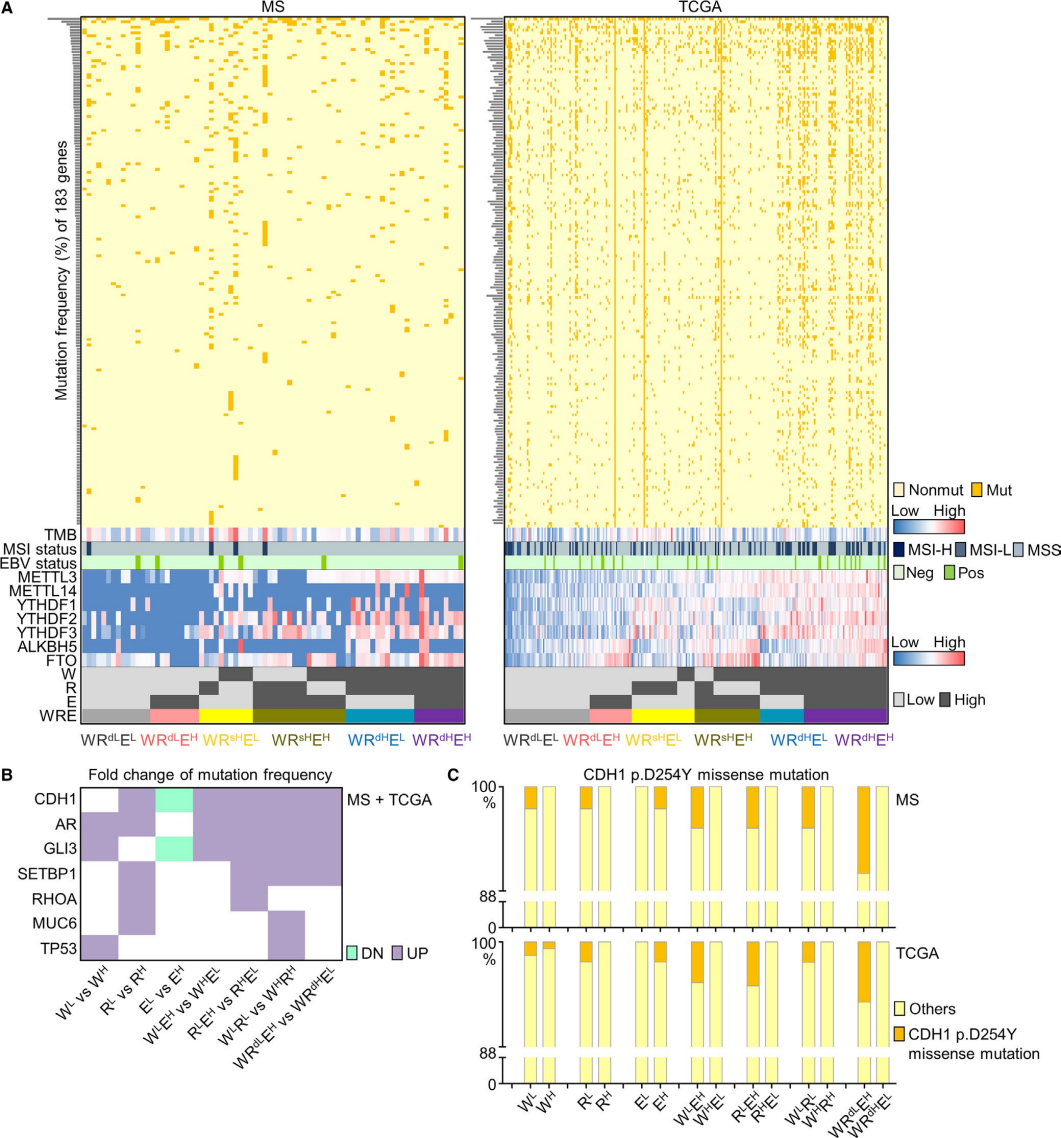
Expressional and mutational landscape of m6A signatures in MS and TCGA datasets. (A) The landscape of 183 high‐frequency mutations and TMB/MSI/EBV status, as well as the distributions of writers, readers, erasers, W/R/E signatures and their double‐ or triple‐stratifications across gastric cancer MS and TCGA datasets were displayed. nonmut, non‐mutated; mut, mutated. MSI‐H, MSI‐high; MSI‐L, MSI‐low. neg, EBV‐negative; pos, EBV‐positive. (B) Mutation frequencies of specific driver genes were compared between low m6A‐indications (W^L^, R^L^, E^H^, W^L^E^H^, R^L^E^H^, W^L^R^L^, WR^dL^E^H^) and high m6A‐indications (W^H^, R^H^, E^L^, W^H^E^L^, R^H^E^L^, W^H^R^H^, WR^dH^E^L^). Fold changes of low‐ vs high‐indications were calculated for both MS and TCGA cohorts and intersected as a heatmap. DN, downregulated mutation rates in low m6A‐indications compared with high m6A‐indications; UP, upregulated mutation rates in low m6A‐indications compared with high m6A‐indications. (C) Frequency of CDH1 p.D254Y missense mutation in patients expressing low or high m6A signatures. EBV, Epstein‐Barr virus; MS, mass‐spectrum; MSI, microsatellite instability; TMB, tumor mutation burden

We then investigated the distribution of the seven m6A regulators and the three generated signatures (W, R, E) across our MS cohort (78 cases) and TCGA GC datasets (289 cases), and assessed their correlations with genomic features. Patients in each cohort were defined as “high” or “low” expressions according to individual levels of W/R/E in a median‐based criterion, and were then double‐stratified by WE/RE/WR or triple‐stratified by WRE signatures (Figure 1A). According to targeted exome sequencing, crucial mutations were assessed across both MS and TCGA cohort. Mutations of METTL3, METTL14, YTHDF1, YTHDF2, YTHDF3, ALKBH5, and FTO were rare, while another 183 genes were found with a mutation frequency higher than 1% in both MS and TCGA cohort (Table S1).

Among the 183 genes, mutation patterns of *CDH1*, *AR*, *GLI3*, *SETBP1*, *RHOA*, *MUC6*, and *TP53* were found closely associated with m6A signatures. For W/R/E single‐stratification, these genes were more frequently mutated in patients with expression of low writer (W^L^), low reader (R^L^), or high eraser (E^H^) signatures. For WE/RE/WR double‐stratifications, their mutation frequencies were higher in W^L^E^H^ than in W^H^E^L^ and higher in R^L^E^H^ than R^H^E^L^, or higher in W^L^R^L^ than in W^H^R^H^ groups. For WRE triple‐stratification, their mutation frequencies were higher in WR^dL^E^H^ than in WR^dH^E^L^ groups. Fold changes of these mutation rates were compared in above stratifications, while a merged plot combining both MS and TCGA datasets was shown (Figure 1B). Since W^L^, R^L^, E^H^, W^L^E^H^, R^L^E^H^, W^L^R^L^, and WR^dL^E^H^ stratifications were defined as low m6A‐indications, while W^H^, R^H^, E^L^, W^H^E^L^, R^H^E^L^, W^H^R^H^, and WR^dH^E^L^ as high m6A‐indications as mentioned in Materials & Method section, these key mutations (*CDH1*, *AR*, *GLI3*, *SETBP1*, *RHOA*, *MUC6*, *TP53*) were collectively associated with reduced m6A. Specifically, a missense mutation (p.D254Y) on *CDH1* was predominantly observed in patients with low m6A‐indications (Figure 1C), suggested key mutations in GC may inhibit m6A modification.

### 3.3 **Reduced m6A predicts adverse outcome in **GC

**Figure 2:**
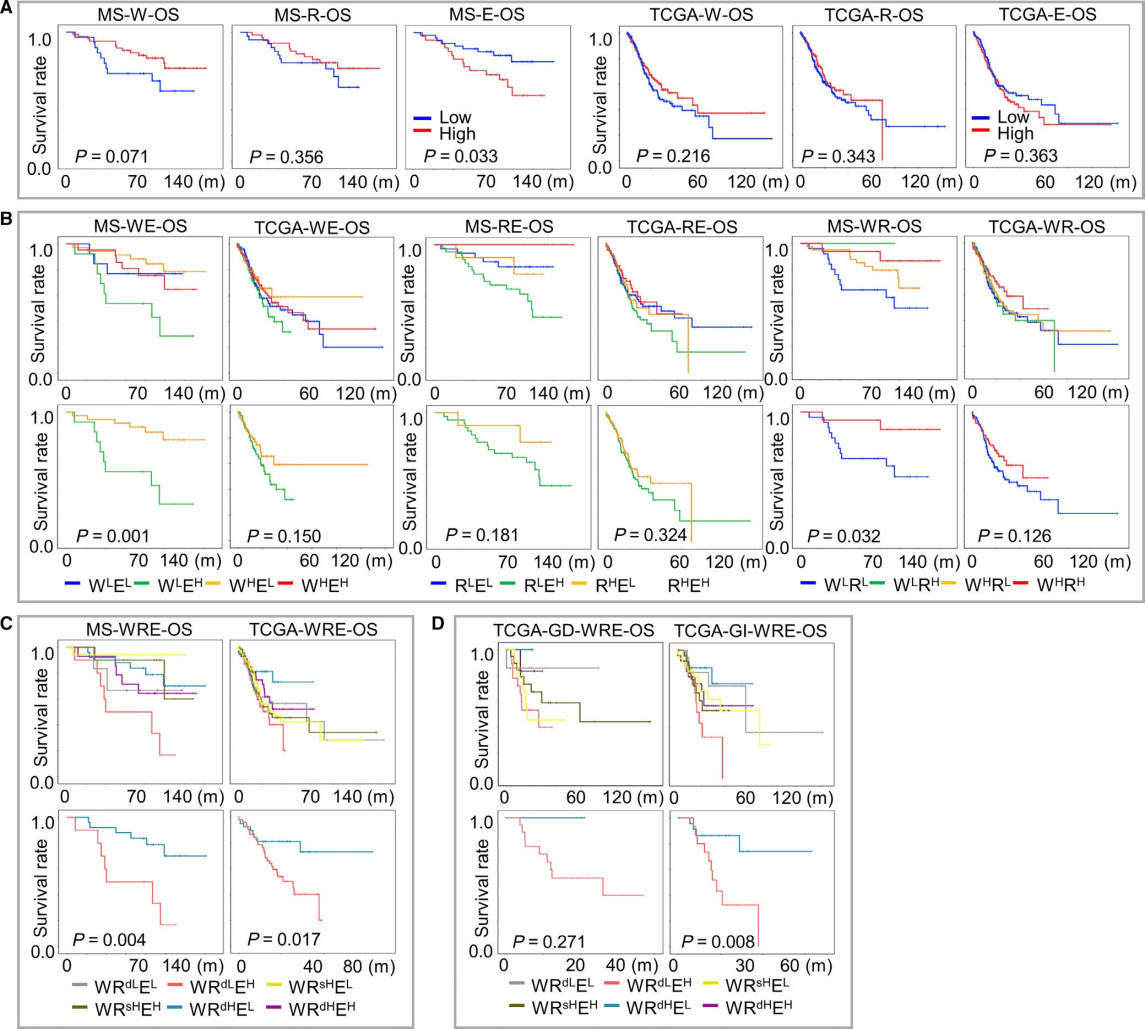
Prognostic implications of m6A signatures in gastric cancer. Overall survival of patients from MS and TCGA cohorts were indicated by (A) W, R and E, or by their (B) double‐ or (C) triple‐stratifications. Survival proportions were assessed by Kaplan‐Meier analysis paired with Log‐rank test. Stratifications with the most diverse m6A‐indications (W^L^E^H^ and W^H^E^L^ in WE, R^L^E^H^ and R^H^E^L^ in RE, W^L^R^L^ and W^H^R^H^ in WR, WR^dL^E^H^ and WR^dH^E^L^ in WRE) were further outlined as individual images. (D) For TCGA cohort, GD (diffuse) and GI (intestinal) patients were separately stratified with WRE signatures to compare survival proportions. MS, mass‐spectrum

According to expression of m6A signatures (W/R/E), GC patients were classified into “high” and “low” expression groups following a median‐based criterion, then assessed by Kaplan‐Meier survival analysis paired with Log‐rank test. Among the three signatures, high expression of E was correlated with adverse OS, while high W predicted prolonged OS despite of insignificant statistics (Figure 2A). Although R's indication for survival was subtle, a favorable prognostic trend for R was also seen in both MS and TCGA datasets (Figure 2A).

Due to that m6A was simultaneously regulated by writers, readers and erasers, pairwise double stratifications for W, R, and E were performed to better reflect m6A regulation. For WE‐stratification, writer‐high‐E‐low (W^H^E^L^) groups displayed the best, while writer‐low‐E‐high (W^L^E^H^) displayed the worst prognosis; similarly, for RE‐stratification, reader‐high‐E‐low (R^H^E^L^) showed more favorable survival than reader‐low‐E‐high (R^L^E^H^). However, for WR‐stratification, writer‐high‐reader‐high (W^H^R^H^) and writer‐low‐reader‐low (W^L^R^L^) groups displayed the most favorable and adverse survival, respectively. Each complete double stratification (Figure 2B, upper panel) and the two most diverse subgroups were demonstrated (Figure 2B, lower panel). Since writers and readers comparably showed favorable prognostic indications, W and R signatures were considered altogether to perform a WRE‐triple stratification. Generally, patients with the highest m6A‐indication in theory (writer and reader‐double‐high‐eraser‐low, WR^dH^E^L^) had significantly improved prognosis than patients with the lowest m6A‐indication in theory (writer and reader double‐low‐eraser‐high, WR^dL^E^H^), while prognosis of the latter ranked as the worst among all WRE stratifications (Figure 2C). Since only diffuse subtype has been enrolled in our MS cohort, we also investigated the prognostic value of m6A‐indications across both diffuse and intestinal subtypes in TCGA cohort. Low m6A‐indications predicted poor survival in both diffuse (GD) and intestinal (GI) subtypes (Figure 2D), suggesting that m6A's impact on prognosis prevalently existed in GC, while expression of m6A regulators and their signatures could be potentially considered as prognostic markers. Moreover, in order to optimize the separation of prognosis, we also selected the best cutoffs with minimum P values for W/R/E signatures by drawing ROC‐curves (Figure S2A). The prognostic tendencies of all stratifications remained unimpaired and the efficiencies of Kaplan‐Meier analysis were generally improved by applying the best cutoffs (Figure S2B‐E).

We also investigated m6A signatures' correlation with clinical variables. Similar to their association to prognosis, low m6A‐indications (W^L^E^H^, R^L^E^H^, W^L^R^L^, and WR^dL^E^H^) were more frequently seen in diffuse subtype and displayed a worse clinical outlook (progressed T/N/M status and advanced tumor stages) than high m6A‐indications (W^H^E^L^, R^H^E^L^, W^H^R^H^, WR^dH^E^L^) in both double‐stratifications (WE/WR/RE, Tables S2 and S3) and triple‐stratifications (WRE, Tables 1 and 2 and Table S4), despite that the statistic diversity was impaired by limited case numbers. The correlation between m6A signatures and patient outcomes indicated that the maintenance of m6A modification suppressed, while reduced m6A modification contributed to carcinogenesis and progression in GC.

**Table 1:** The correlation between triple‐stratification (WRE) of m6A signatures and clinical indexes in MS data

MS	WR^dL^E^H^	WR^dH^E^L^	*P*
Total	10	14	
Gender			0.6785
Male	7 (70)	8 (57)	
Female	3 (30)	6 (43)	
Age			0.6968
≤60	6 (60)	7 (50)	
>60	4 (40)	7 (50)	
T			0.0589
t1	0 (0)	0 (0)	
t2	0 (0)	3 (21)	
t3	4 (40)	7 (50)	
t4	6 (60)	4 (29)	
N			0.2685
n0	1 (10)	5 (36)	
n1	3 (30)	3 (21)	
n2	1 (10)	1 (7)	
n3	5 (50)	5 (36)	
M			—
m0	10 (100)	14 (100)	
m1	0 (0)	0 (0)	
Stage			0.0619
I	0 (0)	2 (14)	
II	2 (20)	5 (36)	
III	7 (70)	7 (50)	
IV	1 (10)	0 (0)	

High m6A‐indication (WR^dH^E^L^) was compared with low m6A‐indication (WR^dL^E^H^). Statistics were performed with Fisher's exact test or Chi‐square test.

Abbreviation: MS, mass‐spectrum.

**Table 2:** The correlation between triple‐stratification (WRE) of m6A signatures and clinical indexes in TCGA cohort

TCGA	WR^dL^E^H^	WR^dH^E^L^	*P*
Total	29	30	
Gender			0.7925
Male	18 (62)	17 (57)	
Female	11 (38)	13 (43)	
Age			0.0391
≤60	11 (38)	4 (13)	
>60	18 (62)	26 (87)	
Lauren			0.0006
Diffuse	13 (45)	2 (7)	
Intestinal	13 (45)	26 (86)	
Others	3 (10)	2 (7)	
T			0.0342
t1	0 (0)	3 (10)	
t2	4 (14)	6 (20)	
t3	20 (69)	9 (30)	
t4	5 (17)	9 (30)	
n/a	0 (0)	3 (10)	
N			0.4169
n0	10 (34)	9 (30)	
n1	4 (14)	4 (13)	
n2	4 (14)	7 (23)	
n3	11 (38)	5 (17)	
n/a	0 (0)	5 (17)	
M			1.0000
m0	27 (93)	26 (86)	
m1	2 (7)	2 (7)	
n/a	0 (0)	2 (7)	
Stage			0.4578
I	3 (10)	6 (20)	
II	11 (38)	6 (20)	
III	13 (45)	9 (30)	
IV	2 (7)	2 (7)	
n/a	0 (0)	7 (23)	

High m6A indication (WR^dH^E^L^) was compared with low m6A indication (WR^dL^E^H^). Statistics were performed with Fisher's exact test or Chi‐square test.

### 3.4 **Reduced m6A promotes proliferation and invasiveness of **GC** cells**

**Figure 3:**
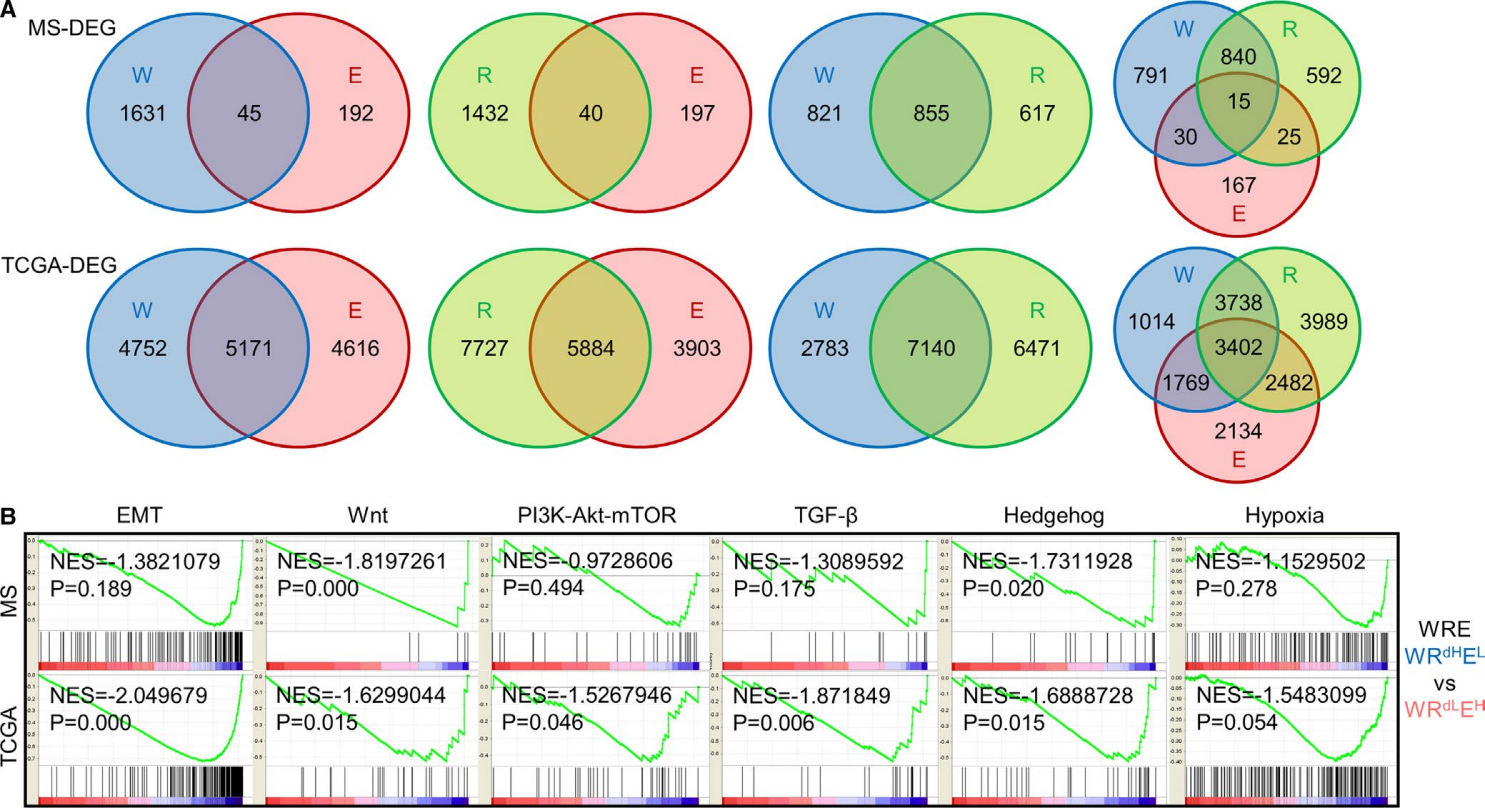
Functional prediction of m6A signatures in gastric cancer. (A) DEGs were generated by comparing patients harboring high‐m6A signatures with patients harboring low‐m6A signatures (W‐high vs W‐low, R‐high vs R‐low, E‐high vs E‐low), then numerated and plotted as Venn diagrams. (B) H1 and C6 gene sets were enriched between WR^dH^E^L^ (highest m6A‐indication) and WR^dL^E^H^ (lowest m6A‐indication) groups in WRE triple‐stratifications. The six gene sets with the highest enrichment scores (EMT, Wnt, PI3K‐Akt‐mTOR, TGF‐β, Hedgehog, Hypoxia) were displayed. DEGs, differentially expressed genes; EMT, epithelial‐mesenchymal transition; NES, normalized enrichment score

In order to elucidate m6A modification's biological association with GC, we performed pathway analysis for m6A signatures. The number of overlapping DEGs (differentially expressed genes) between W and R (855/7140 for MS/TCGA) were much larger than between W and E (45/5171 for MS/TCGA) or between R and E (40/5884 for MS/TCGA) (Figure 3A), suggesting that writers and readers shared a higher compatibility in modulating biological events than each of them shared with erasers. GSEA was performed for W/R/E stratifications in MS and TCGA cohorts. Hallmark (H) and Oncogenic (C6), the two gene sets that majorly defining cancer‐related pathways and phenotypes, were used for analysis. Double stratifications exhibited that high m6A‐indications (W^H^E^L^, R^H^E^L^, W^H^R^H^) were negatively enriched in multiple oncogenic phenotypes/pathways, including EMT, Wnt, PI3K‐Akt‐mTOR, TGF‐β, Hedgehog and hypoxia‐related genes in both MS and TCGA data (Figure S3A‐C). Similarly, for triple stratification, WR^dH^E^L^ group (highest m6A‐indication) was also negatively enriched in these oncogenic phenotypes/pathways (Figure 3B), implicating that m6A potentially represses these signaling and inhibits corresponding phenotypes.

**Figure 4:**
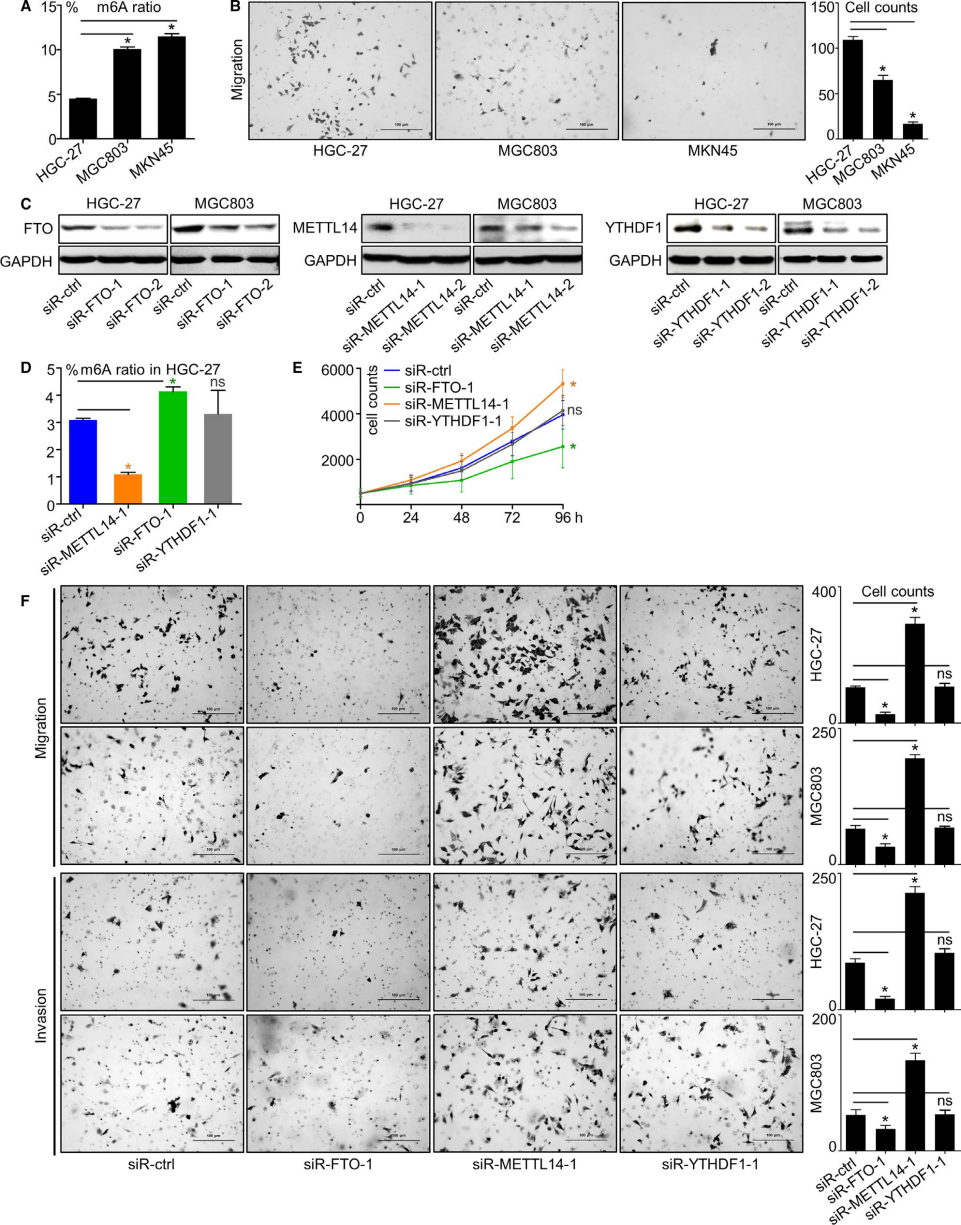
Reduced m6A enhanced proliferation and invasiveness in GC cells. (A) Proportions of m6A modification in total‐RNA and (B) migration capabilities of GC cell lines HGC‐27, MGC803 and MKN45 were assessed. (C) Respective changes of METT14, FTO and YTHDF1 protein expressions in HGC‐27 and MGC803 after being knocked‐down by siRNA. (D) m6A proportions in total‐RNA and (E) proliferation rates of HGC‐27 after METT14, FTO or YTHDF1 knockdown were displayed. (F) Migration and invasion capabilities of HGC‐27 and MGC803 after METT14, FTO or YTHDF1 knockdown were assessed. *, *P* < 0.05. ns, not significant. GC, gastric cancer

For verification, we assessed the total level of m6A modification in multiple GC cell lines. The content of RNA bearing m6A modification increased in cascade from HGC‐27, MGC803 to MKN45 (Figure 4A). Meanwhile, the migration capability decreased from HGC‐27, MGC803 to MKN45 (Figure 4B), giving hint that m6A may negatively affect GC cells' invasiveness. Among the seven regulators, by weighing each genes' abundance and mechanistic details in modulating m6A modification, we selected METTL14, YTHDF1, and FTO, respectively, as the representative writer, reader and eraser for following in vitro experiments.

Expressions of these three genes were effectively repressed by two individual siRNA sequences (Figure 4C), and the most effective sequences were used for further analysis. The impact of regulators on m6A modification was investigated. In HGC‐27, ratio of m6A modification in total RNA was downregulated by METTL14 knockdown, upregulated by FTO knockdown and unaffected by YTHDF1 knockdown (Figure 4D), verifying the fact that writers promote while erasers suppress m6A modification, also emphasizing that the degree of m6A modification could be represented by combination of W/R/E signatures. Phenotypically, proliferation of HGC‐27 cell was enhanced by METTL14 knockdown or repressed by FTO knockdown, while knockdown of YTHDF1 exerted minimal impacts on proliferation (Figure 4E). Similar to proliferation, GC cells' migration and invasion capabilities were strengthened by METTL14 knockdown or inhibited by FTO knockdown, yet largely unaffected by YTHDF1 knockdown (Figure 4F).

### 3.5 m6A antagonizes Wnt and PI3K‐Akt signaling

**Figure 5:**
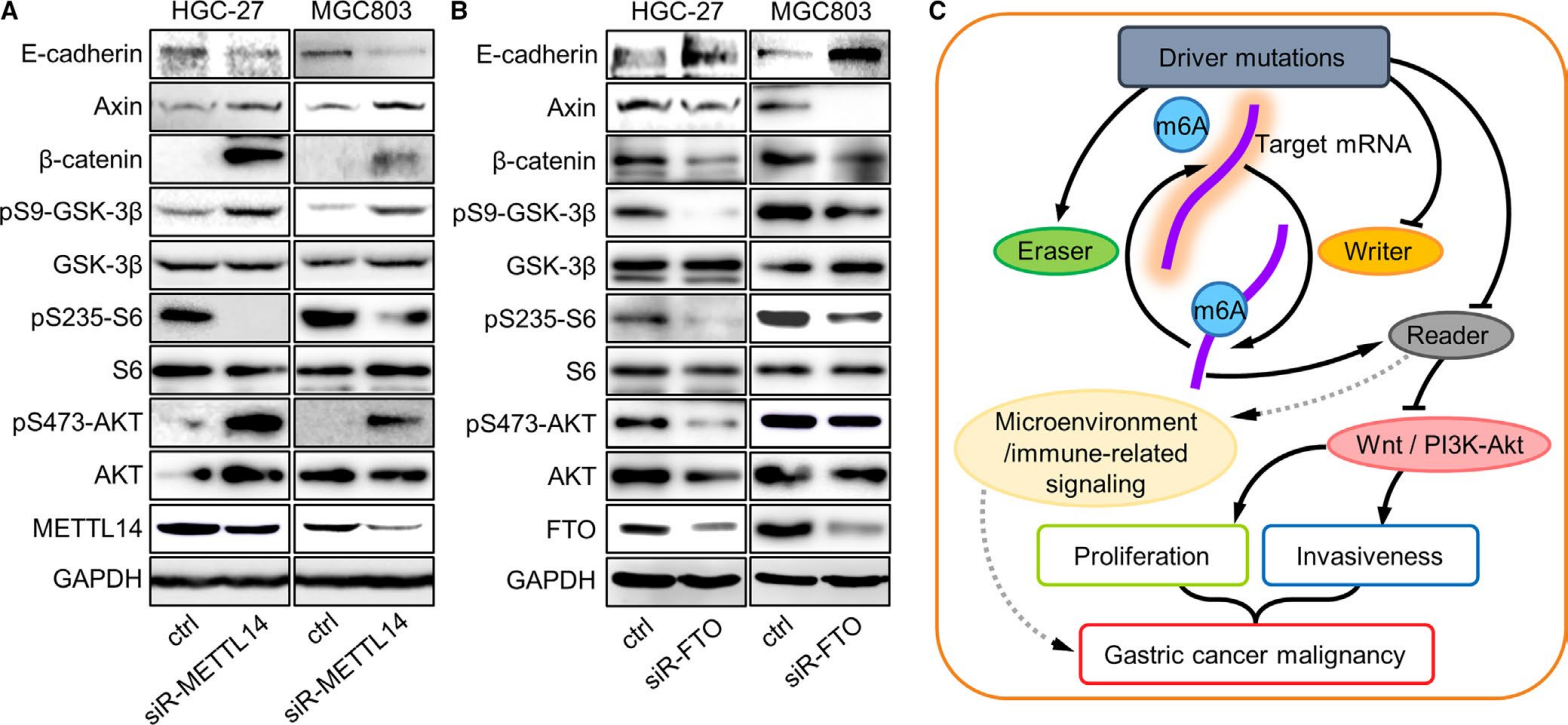
Reduced m6A activated Wnt and PI3K‐Akt signaling in GC cells. After (A) METTL14 or (B) FTO knockdown, changes of major components in Wnt and PI3K‐Akt pathways were assessed by western blot. (C) A hypothetical outline of m6A's roles in GC progression through its interactions with upstream or downstream signaling networks. GC, gastric cancer

Among m6A regulators, readers (YTHDF1) seemed to be the least effective category in inducing phenotypical changes. As a consequence, we selected only the representative molecules for writers (METTL14) and erasers (FTO) to assess the signaling pathways previously predicted to be related to m6A modification. In line with GSEA results, Wnt (marked by β‐catenin/Axin1 expression and Ser9‐GSK‐3β phosphorylation) and PI3K‐Akt (marked by Ser473‐Akt and Ser235‐S6 phosphorylation) signaling were activated by METTL14 knockdown (Figure 5A), or inhibited by FTO knockdown (Figure 5B). Conversely, expression of E‐cadherin (*CDH1*) was downregulated by METTL14 knockdown, or upregulated by FTO knockdown in HGC‐27 and MGC803 cells (Figure 5A,B). As a tumor suppressive factor and a marker for epithelial phenotype, E‐cadherin changes elicited by METTL14/FTO knockdown were in line with the activation/inhibition of Wnt/PI3K‐Akt pathways.

Since results from investigating two distinct m6A regulators were in consistent (ie, proliferation/invasiveness and Wnt/PI3K‐Akt signaling were enhanced by knockdown of m6A writer gene METTL14, or suppressed by knockdown of m6A eraser gene FTO), it is reasonable to believe that the carcinogenic phenotypes and activated signaling were more likely to be controlled by regulator‐mediated m6A changes, rather than directly by m6A regulator genes. Taken together, m6A modification plays a tumor suppressive role in GC, probably through repressing Wnt and PI3K‐Akt signaling. Considering the correlation between mutations and low m6A‐indication signatures, the content and functions of m6A in GC might be impaired by specific mutations. An m6A related network in regulating GC progression was outlined (Figure 5C).

### 3.6 m6A modification was potentially correlated with immunotherapy features and interferon signaling

Immunotherapy is currently one the most promising approaches for anti‐cancer treatment.^27^, ^28^ Due to that tumor mutation burden (TMB), microsatellite instability (MSI), and Epstein‐Barr virus (EBV) were recognized as markers for cancer immunotherapy, we investigated m6A regulators' correlations with these parameters (Figure 1A,B). Expressions of all m6A regulators were insignificantly differed upon EBV infection, while writers or readers were largely irrelevant to TMB or MSI status. Nevertheless, TMB level and MSI ratio were significant higher in patients with low expressions of ALKBH5, FTO, or E signature (Figure S4A,B), suggesting m6A modification was positively correlated with TMB/MSI status, and might be involved in immune responses of GC.

Recent studies pointed out that m6A modification hinders cellular response to virus infection by repressing type I interferon synthesis.^29^, ^30^ Since interferons were also reported tightly related to tumor microenvironment, immune checkpoints, and immunotherapy responses,^31^ we explored m6A's impact on interferon signaling of GC. We performed m6A‐based enrichment analysis for interferon related gene sets. Type I interferon, interferon α/β and interferon γ signaling gene sets displayed negative trends of enrichment in high‐m6A stratifications (W^H^E^L^, R^H^E^L^, W^H^R^H^, WR^dH^E^L^) (Figure S4C). For verification, we testified the expressions of IFNs and ISG15 mRNAs in siRNA‐treated cells with qPCR. Transcript levels of IFNA/IFNB/IFNG/ISG15 were upregulated by METTL14/YTHDF1 knockdown in both AGS and HGC27 cells (Figure S4D). While according to ELISA assay, the levels of secreted interferon α/β/γ proteins in HGC27 were slightly upregulated by METTL14/YTHDF1 knockdown, or downregulated by FTO knockdown (Figure S4E), which was in accordance with the findings of Winkler et al.^29^ Since interferons exerted dual functions in mediating cancer immunity, m6A may modulate immune responses of GC through repressing interferon production (Figure 5C). However, due to the illegibility of current data, whether RNA m6A modification regulates interferon/immune response of GC remains to be validated, and m6A's linkage with immunotherapy deserved further investigation.

## 4 DISCUSSION

Methylation plays important roles in multiple biological and pathological processes, including cancer. Methylation was conventionally recognized to modulate DNA unwinding and transcription, while with the understanding of epigenetic control deepened, the cognition of methylation on nucleic acid has been expanded from DNA to RNA. As the most prevalent epigenetic modification on eukaryotic RNA, m6A is specified by the methylation of adenosine and is controlled by writers, readers, and erasers. The degree of m6A modification is enhanced by writers or reduced by erasers, while despite that readers do not directly influence m6A level, they recognize and exert the effects of m6A modifications. RNA m6A modification has been reported to influence carcinogenesis particularly in AML and glioblastoma, yet its roles in regulating GC carcinogenesis and progression remained to be elucidated. In this study, by combining DNA, RNA, and protein‐based omics study and in vitro experiments, we assessed the expressions of writers/readers/erasers and their implications in GC.

With the advance of research, the panel of genes reported to be involved in m6A regulation was in rapid expansion.^32^, ^33^ Considering the roles of other emerging genes in modulating m6A has not been well characterized yet, only the seven canonical regulators (METTL3, METTL14, YTHDF1, YTHDF2, YTHDF3, ALKBH5, FTO) were included in our study. Since the clinicopathological relevance of the seven m6A regulators on single gene level were statistically insignificant, they were functionally categorized and merged as W, R, and E signatures to better represent expressions of writers, readers, and erasers. Our analysis in both protein (MS) and mRNA (TCGA) datasets suggested that writers and readers were tumor suppressive while erasers play a tumorigenic role in GC, however, controversial roles of METTL3/YTHDF2 in other cancer types were reported. METTL3 was found highly expressed in hepatocellular cancer (HCC), whose overexpression elicited HCC growth and lung metastasis via modulating level of SOCS2 in a YTHDF2‐dependent manner.^10^ Furthermore, METTL14 displayed higher methyltransferase activity than METTL3 in vitro.^6^, ^7^ As a consequence, although the positive correlation with favorable clinical indications and prognosis implicated both METTL3/METTL14's tumor suppressive role in GC, METTL14, instead of METTL3, was selected as the representative writer for in vitro verifications. Due to similar reason, YTHDF1 was selected as the representative reader instead of YTHDF2. Furthermore, according to our MS cohort, the protein level of FTO was much higher than ALKBH5 in GC tissues, thus we chose FTO over ALKBH5 as the representative eraser. Elisa assay performed specifically on RNA exhibited that m6A level was reduced by knockdown of METTL14 and enhanced by knockdown of FTO, validating the changes of m6A modification can be reflected by alterations of m6A regulators. Since readers recognize the writer‐imprinted m6A and transduce signals, we classified high‐expressions of W or/and R, along with low expressions of E, as symbols for augmented m6A effects despite that YTHDF1 knockdown elicited minimal changes to m6A level.

Analysis in MS and TCGA cohort demonstrated that the correlations of W with clinicopathological features (prognosis/TNM/stage) were opposite to E (W^H^E^L^ displayed significantly improved outcome than W^L^E^H^) and were strengthened by R (W^H^R^H^ displayed significantly improved outcome than W^L^R^L^), while WRE triple‐stratification augmented the diversity (WR^dH^E^L^ displayed significantly improved outcome than WR^dL^E^H^). This consistency that writers and readers shared argued for the notion that they were both indicators for high‐m6A, and m6A plays a tumor suppressive role in GC. Importantly, results acquired from analyzing MS data (DNA and protein) were in accordance with results from TCGA data (DNA and RNA), while m6A consistently predicted favorable prognosis in both diffuse and intestinal GC, supporting the notion that disturbed expression of m6A regulators was a common phenomenon in GC and exerted similar clinicopathological functions across different GC subtypes.

Tumor mutation burden and MSI ratio were specifically higher in patients expressing low eraser genes/signatures, yet more evidences are required to elucidate their mechanistic correlation with m6A. Since TMB and MSI has been considered as instructive features for anti‐cancer immunotherapy, m6A's impacts and implications on immune checkpoints, microenvironments and tumor immune response in GC deserve further exploration.^34^, ^35^ Interferons belong to the cytokine family and were originally recognized as promising agents against infection and cancer, nonetheless, recent studies demonstrated that type I/II interferons also impaired body's defense against cancer and facilitated tumor growth by stimulating diverse interferon‐stimulated genes.^31^, ^36^ Interferons accelerated invasiveness of certain types of cancer, and promoted their resistance to NK (natural killer) cells.^37^ Interferons directed the up‐regulation of checkpoint molecule PD‐L1 and immunosuppressive metabolite IDO in tumor/myeloid cells, thereby establishing a microenvironment that compromise the effectiveness of anti‐tumor T cells and induced resistance to immunotherapy.^37^, ^38^, ^39^ Winkler and colleagues demonstrated that deletion of METTL3 (m6A writer) and YTHDF2 (reader) stabilized the mRNA of IFNB,^29^ while Rubio et al reported that depleting METTL14 (writer)/ALKBH5 (eraser) increased/reduced IFNB mRNA production, respectively.^30^ Our work also indicated that type I (α, β) and type II (γ) interferons might be negatively regulated by RNA m6A methylation, thus we inferred that RNA m6A modification may participate in the formation of GC's tumor microenvironment and responses to immunotherapy through mediating repressions of interferon signaling. Nevertheless, it is noteworthy that m6A's linkage with interferons were inferior to PI3K‐Akt or Wnt pathways as shown by both in silico and in vitro experiments, suggesting that m6A may share indirect connections with interferons. Considering the two‐faced role of interferons in cancerous immunity, the mutual relationships and crosstalk between RNA m6A modification and interferons demand verification and exploration.

In GC cell lines, levels of m6A were evidently repressed under METTL14 or enhanced under FTO knockdown, while proliferation and migration/invasion changed oppositely to m6A levels, verifying that m6A antagonizes GC malignancy. Since m6A level was largely unaffected by YTHDF1 knockdown, it is reasonable that as signal transducers, readers (YTHDF1) were less prominent in affecting proliferation or invasiveness changes than writers. As a consequence, only writer and eraser (represented by METTL14 and FTO, respectively)'s impacts to GSEA‐predicted pathways were further investigated by western blot assay. As shown by vitro investigations, Wnt and PI3K‐Akt pathways were activated by METTL14 knockdown and repressed by FTO knockdown; on the contrary, E‐cadherin was downregulated by METTL14 knockdown or upregulated by FTO knockdown. The fact that loss of METTL14 elicited opposite phenotypical and molecular changes to loss of FTO proved that writers and erasers are functionally complementary in GC, thus it can be ruled out that m6A regulators influence malignancy via additional mechanisms other than regulating m6A. Nonetheless, the molecular events controlled by m6A modification require following elucidation.

Although GC does not carry as much Wnt signaling‐related mutations as intestinal cancer (~80%), certain genetic alterations are frequently observed in GC.^40^, ^41^, ^42^, ^43^ PI3K‐Akt signaling is inappropriately altered and activated by various genetic abnormalities, including mutations (*PIK3CA*), overexpression or amplifications (*PIK3CA*, *AKT1*, *MET*), and loss of suppressors (*PTEN*).^44^ As a major constituent of cell adhesion machinery, E‐cadherin (*CDH1*) is also prevalently found deregulated or mutated in GC, which is more frequently observed in diffuse than in intestinal subtypes.^45^, ^46^ Apart from mutation or expression abnormalities, Wnt, PI3K‐Akt as well as E‐cadherin are tightly controlled by DNA methylation. Recent studies demonstrated that epigenetic activation of Wnt signaling (marked by enhanced β‐catenin and silent RNF43) is prevalently observed in multiple types of cancer.^47^, ^48^, ^49^ Reduced methylation of *PTEN* CpG islands suppresses, while hyper‐methylation of *PTEN* CpG islands stimulates PI3K‐Akt signaling.^50^, ^51^ Methylation of *CDH1* CpG islands also increases in malignant tissues and contributes to tumorigenesis.^52^, ^53^ Nevertheless, RNA methylation is merely reported to be involved with these pathways. To the best of our knowledge, it is the first time to reveal that RNA m6A modification impacts Wnt/PI3K‐Akt signaling and expression of E‐cadherin. Wnt and PI3K‐Akt signaling as well as their downstream networks control the majority of cancer hallmarks, including cell cycle, proliferation, survival, motility, angiogenesis, and drug resistance.^54^, ^55^ Conversely, strong anti‐metastatic roles of E‐cadherin were reported in multiple types of cancer, while patients carrying *CDH1* mutations or loss of E‐cadherin expression displayed poor survival than non‐mutated patients.^56^, ^57^ Membrane β‐catenin links E‐cadherin to the cytoskeleton as an integral structural of adherence junctions, while activation of Wnt signaling breaks the anchorage and promotes the accumulation of β‐catenin in nucleus, thus activating a series of signaling cascades and eliciting EMT.^43^, ^58^ PI3K‐Akt signaling also induces E‐cadherin downregulation via mTOR or MAPK cascade.^59^ Due to the fact that Wnt/PI3K‐Akt signaling cascades are tightly linked to each other and both promote EMT by downregulating E‐cadherin, we hypothesized that high‐invasiveness phenotypes under low m6A‐indications were induced via Wnt‐ and/or PI3K‐Akt‐dependent E‐cadherin inhibition, probably regulated by loss of m6A on Wnt/PI3K‐Akt signaling components.^56^, ^60^ However, the existence of this hypothetic signaling axis remains to be verified, and whether m6A regulates E‐cadherin expression through other molecules/pathways (such as TGF‐β, hedgehog, and hypoxia) also deserves further exploration.

Among genomic aberrances, mutations of *CDH1*, *AR*, *CLI3*, *SETBP1*, *RHOA*, *MUC6*, and *TP53* were more frequently observed in low m6A‐indication groups. Although specific mutations on *CDH1* does disrupt splicing, reduce the protein half‐life and impair the functions of E‐cadherin, molecular and pathological implications of the p.D254Y missense mutation majorly observed in low‐m6A GC patients remains uncategorized and unreported.^61^, ^62^ Opposite to the tumor‐suppressive E‐cadherin, AR (Androgen receptor) drives carcinogenesis by boosting Wnt/β‐catenin signaling, whose activating mutations and amplification are specifically more abundant in prostate cancer.^63^, ^64^ GLI3 is a transcription factor that negatively modulates hedgehog signaling, yet its role in GC remains unclear.^65^ SETBP1 forms a heterodimer with SET protein and inhibited the tumor suppressive functions of PP2A, *SETBP1* mutation is detected in AML and non‐small cell lung cancer, which generally blocks its ubiquitination and elicits abnormal high expression.^66^, ^67^, ^68^ RHOA is a GTPases that influences multiple biological processes, upregulation of RHOA is associated with tumorigenesis, while instead of intestinal subtype, *RHOA* mutation is specifically identified in diffuse subtype of GC.^69^, ^70^ MUC6 encodes a secretory mucin and protects gastric mucosa, whose reduction inclines chronic mucosal injury and promotes carcinogenesis. Ratio of driver mutations on *MUC6* is higher in MSI than MSS GC.^70^, ^71^, ^72^ Notably, apart from *TP53*, none of these mutated genes has been reported to be associated with m6A. In AML patients, mutations and CNVs of m6A regulators are associated with the presence of *TP53* mutations.^18^ Now that epigenetic modifications are unlikely to induce genomic alterations, we inferred that changes of m6A regulators as well as corresponding molecular/phenotypical events in GC were elicited by upstream mutations, probably through activation of cancer driver genes or loss of tumor suppressor genes.

In conclusion, we inferred that GC progression might be preferentially promoted by m6A‐loss‐mediated activation of oncogenic signaling (such as Wnt and PI3K‐Akt), or by m6A‐gain‐mediated repression of tumor suppressive signaling. As the first attempt to systemically elucidate m6A's clinicopathological role in GC, our work provides insights into the tumor suppressive function of m6A and its potential molecular mechanisms. However, m6A's relationship with specific gene mutations, other oncogenic pathways and its functional details in controlling tumorigenesis/progression merit further investigation.

## CONFLICT OF INTEREST

The authors report no conflicts of interest.

## Data Availability

The data that support the findings of this study are openly available in TCGA (https://portal.gdc.cancer.gov/) and reference number.^25^
