# Supplementary figures and images for: Reduced m6A modification predicts malignant phenotypes and augmented Wnt/PI3K‐Akt signaling in gastric cancer
Source: Cancer Med. 2019 Jun 26;8(10):4766–81. doi: 10.1002/cam4.2360 (PMC6712480; doi:10.1002/cam4.2360)

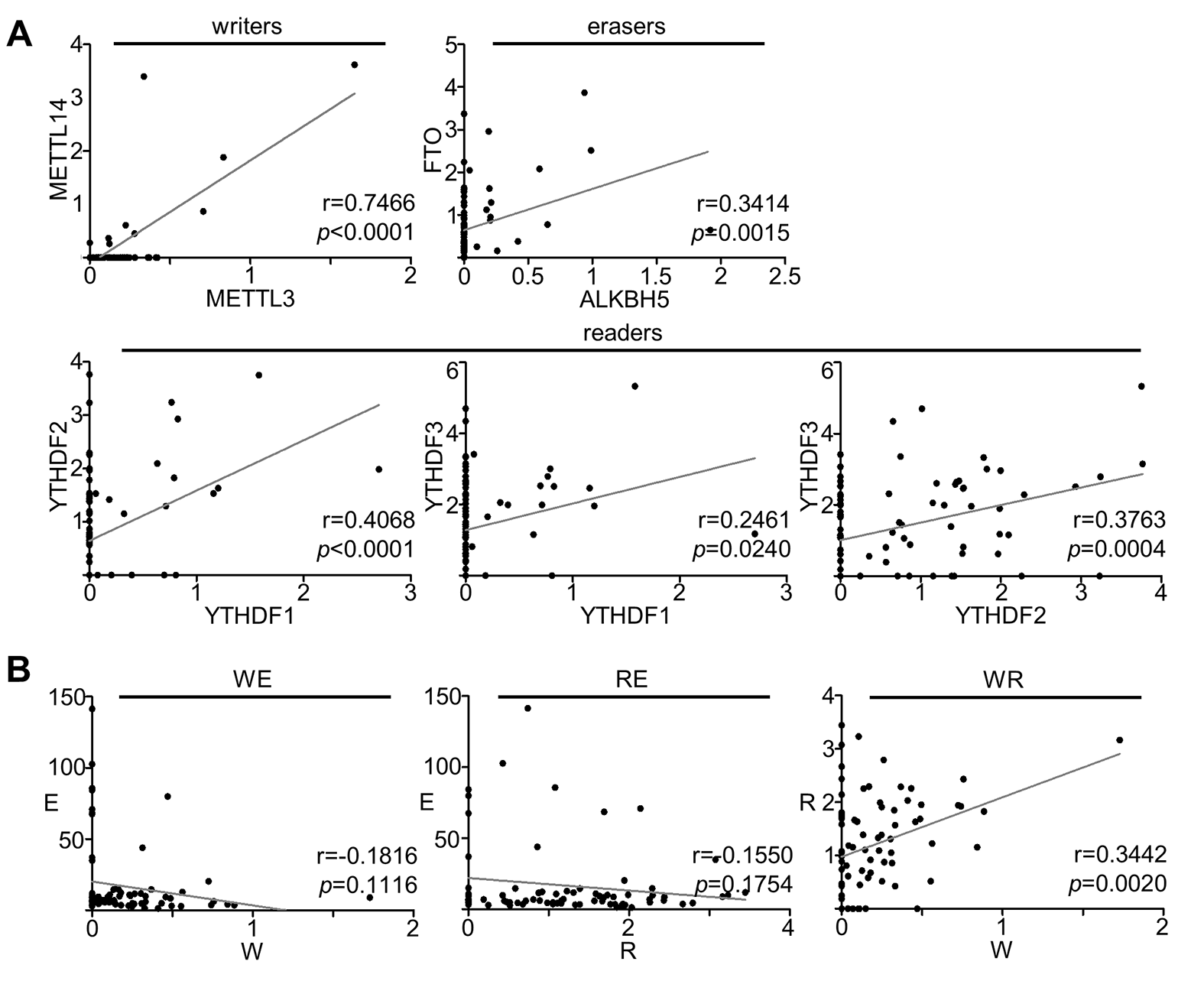

Supplement: Supplementary file 1 [file CAM4-8-4766-s001.tif]

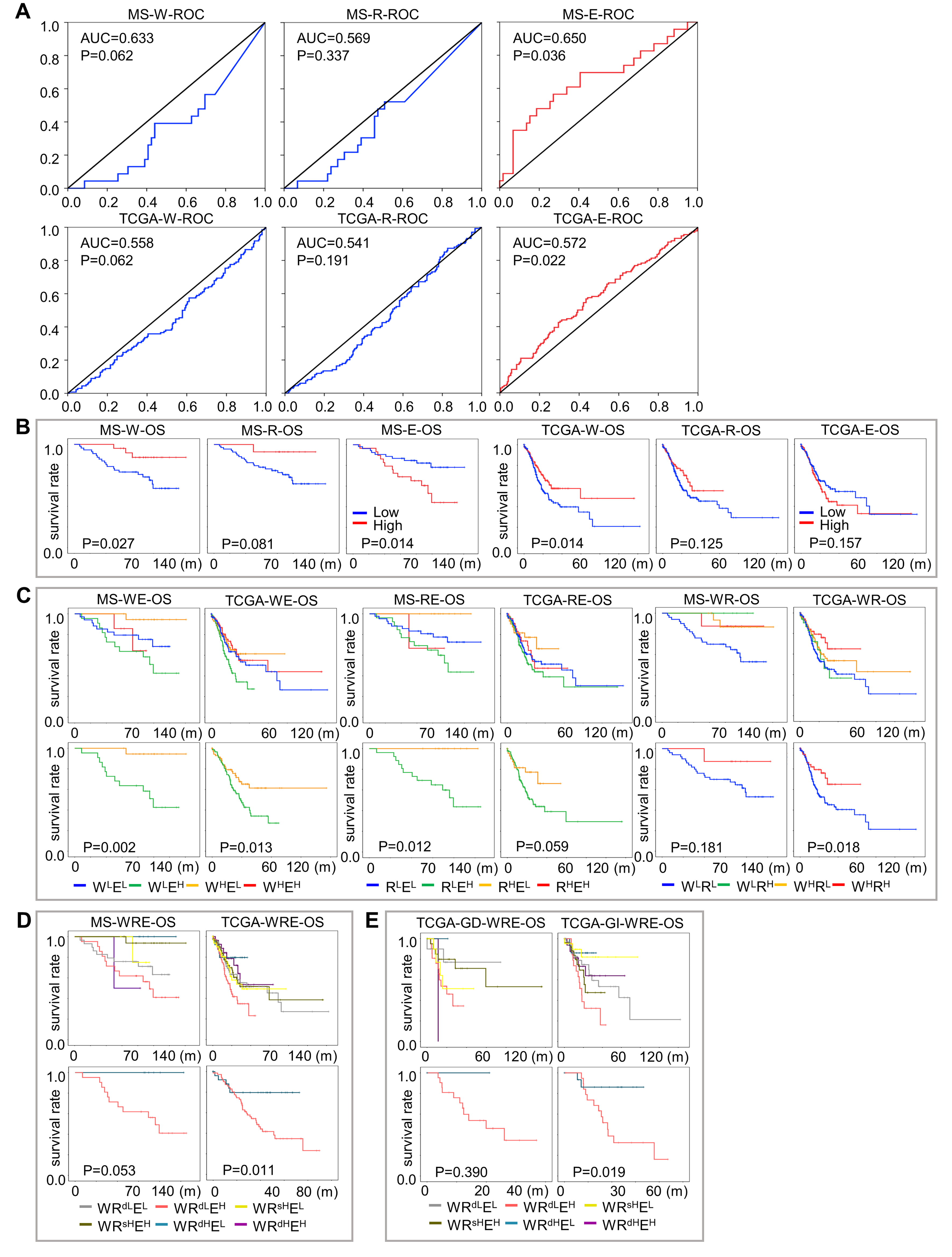

Supplement: Supplementary file 2 [file CAM4-8-4766-s002.tif]

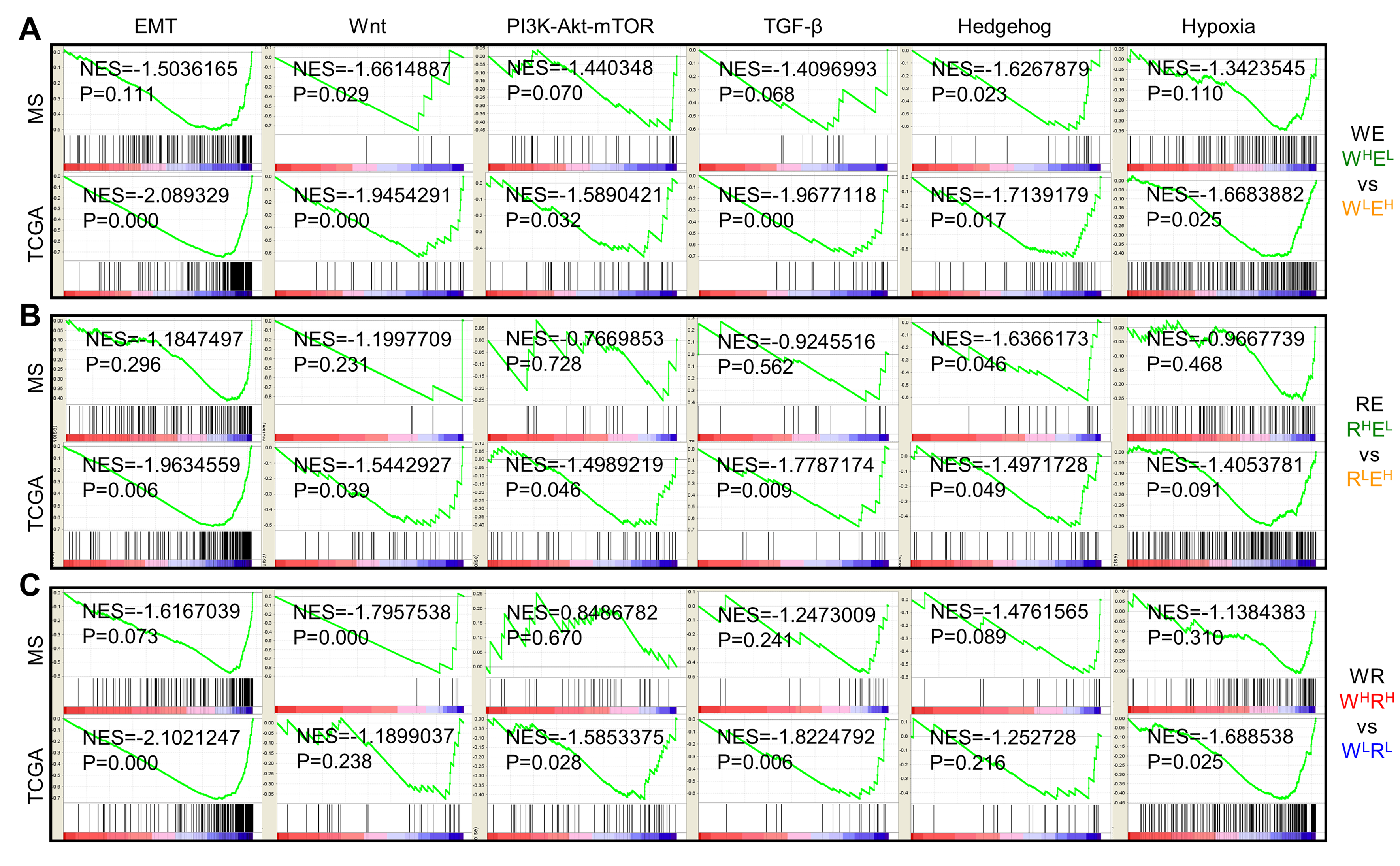

Supplement: Supplementary file 3 [file CAM4-8-4766-s003.tif]

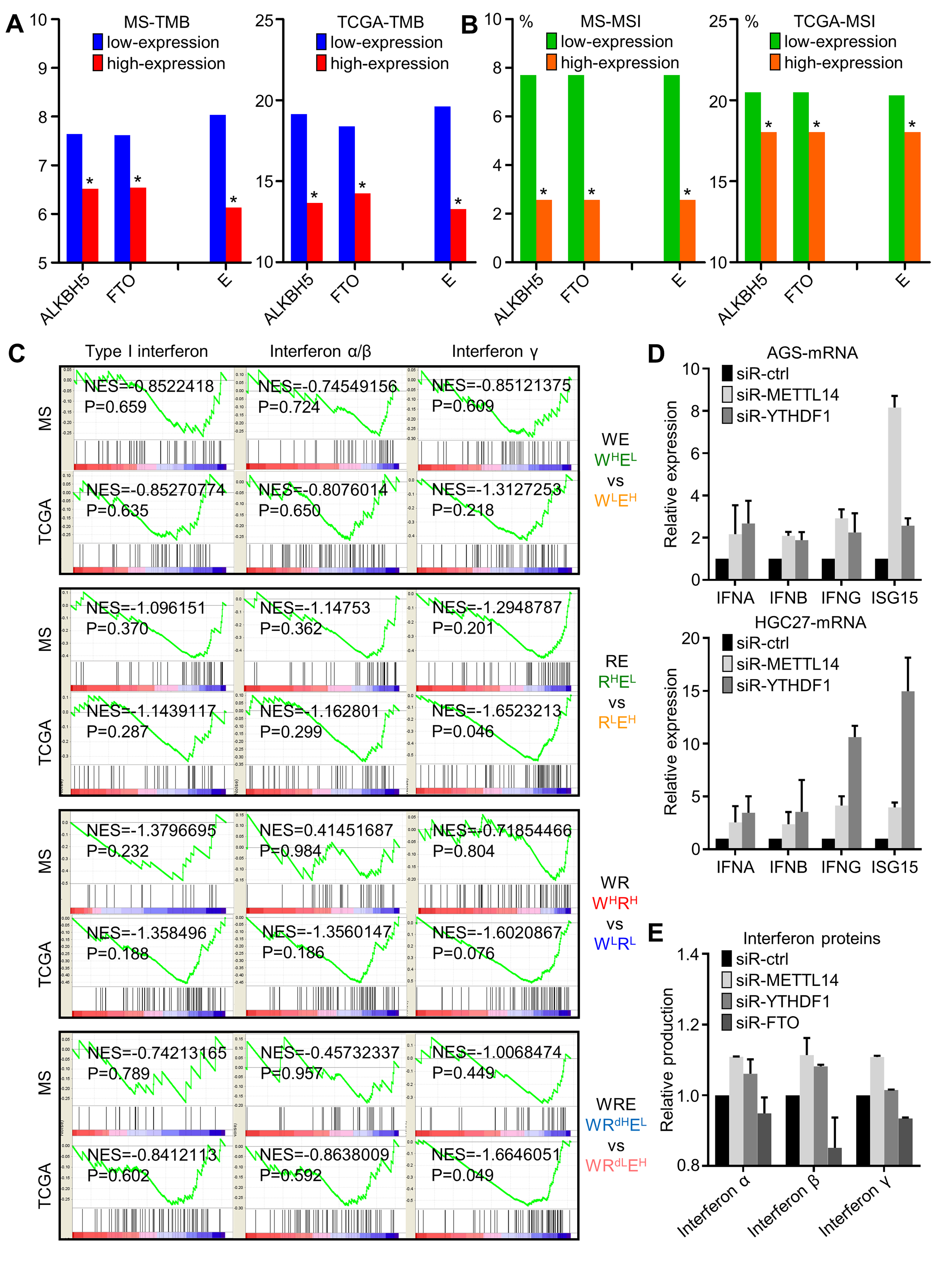

Supplement: Supplementary file 4 [file CAM4-8-4766-s004.tif]
