# Supplementary material for: Reduced m6A modification predicts malignant phenotypes and augmented Wnt/PI3K‐Akt signaling in gastric cancer
Source: Cancer Med. 2019 Jun 26;8(10):4766–81. doi: 10.1002/cam4.2360 (PMC6712480; doi:10.1002/cam4.2360)
Supplement: Supplementary file 5 [file CAM4-8-4766-s005.docx]

**Supplementary Tables**

**Table S1.** A list of 183 genes and their mutation frequencies in gastric cancer MS and TCGA cohorts. The seven mutated genes correlated with m6A signatures were highlighted in bold.

|  | mutation frequency (%) | |  |  | mutation frequency (%) | |  |  | mutation frequency (%) | |
| --- | --- | --- | --- | --- | --- | --- | --- | --- | --- | --- |
| Gene ID | MS | TCGA |  | Gene ID | MS | TCGA |  | Gene ID | MS | TCGA |
| ***TP53*** | 50 | 49.13495 |  | *PLEKHA6* | 3.846154 | 8.650519 |  | *PRKAR1A* | 1.282051 | 2.768166 |
| ***CDH1*** | 26.92308 | 13.14879 |  | *BRAF* | 3.846154 | 11.76471 |  | *JAK2* | 1.282051 | 7.958478 |
| *KMT2D* | 17.94872 | 22.14533 |  | *TMPO* | 3.846154 | 6.228374 |  | *BCL10* | 1.282051 | 0.692042 |
| *ARID1A* | 12.82051 | 31.14187 |  | *MSH2* | 3.846154 | 4.49827 |  | *GOT2* | 1.282051 | 2.768166 |
| *FAT4* | 12.82051 | 30.10381 |  | *IKZF1* | 3.846154 | 5.536332 |  | *MYC* | 1.282051 | 4.49827 |
| *SPTA1* | 12.82051 | 25.95156 |  | *NF1* | 3.846154 | 19.03114 |  | *HIST1H3B* | 1.282051 | 1.038062 |
| ***RHOA*** | 12.82051 | 7.266436 |  | *ERBB3* | 3.846154 | 16.26298 |  | *PIK3R1* | 1.282051 | 4.152249 |
| *APC* | 11.53846 | 18.3391 |  | *ERBB2* | 3.846154 | 7.266436 |  | *B2M* | 1.282051 | 8.99654 |
| *RIMS2* | 10.25641 | 26.6436 |  | *KIF2B* | 3.846154 | 10.72664 |  | *TBL1XR1* | 1.282051 | 5.190311 |
| *PIK3CA* | 10.25641 | 24.56747 |  | *DDX3X* | 3.846154 | 7.612457 |  | *AGO4* | 1.282051 | 7.612457 |
| *DNAH7* | 8.974359 | 21.79931 |  | *ACVR2A* | 3.846154 | 5.190311 |  | *IDH2* | 1.282051 | 1.730104 |
| *PKHD1* | 8.974359 | 20.41522 |  | *VIM* | 2.564103 | 6.574394 |  | *FLT3* | 1.282051 | 5.190311 |
| *KMT2C* | 8.974359 | 26.98962 |  | *FUBP1* | 2.564103 | 7.612457 |  | *CDC73* | 1.282051 | 8.650519 |
| *BRCA2* | 7.692308 | 13.49481 |  | *IMPG2* | 2.564103 | 12.45675 |  | *MYD88* | 1.282051 | 3.114187 |
| *ATM* | 7.692308 | 20.0692 |  | *ZBTB20* | 2.564103 | 13.14879 |  | *HLA-B* | 1.282051 | 7.266436 |
| ***MUC6*** | 7.692308 | 15.57093 |  | *RAD21* | 2.564103 | 5.882353 |  | *BCLAF1* | 1.282051 | 5.190311 |
| *BAP1* | 7.692308 | 5.536332 |  | *ETV6* | 2.564103 | 5.190311 |  | *MYEOV* | 1.282051 | 2.768166 |
| *FBXW7* | 6.410256 | 11.07266 |  | *SNRPN* | 2.564103 | 8.304498 |  | *SERPINB8* | 1.282051 | 4.49827 |
| *KRAS* | 6.410256 | 10.0346 |  | *BCOR* | 2.564103 | 10.72664 |  | *SMC1A* | 1.282051 | 4.49827 |
| *CREBBP* | 6.410256 | 15.57093 |  | *CEBPZ* | 2.564103 | 6.920415 |  | *FGFR2* | 1.282051 | 8.650519 |
| *RPL22* | 6.410256 | 1.038062 |  | *BAX* | 2.564103 | 2.768166 |  | *PGM5* | 1.282051 | 8.99654 |
| *LMAN1* | 6.410256 | 9.342561 |  | *PTPN23* | 2.564103 | 8.650519 |  | *FOXL2* | 1.282051 | 2.422145 |
| ***AR*** | 6.410256 | 9.342561 |  | *FOXC1* | 2.564103 | 1.038062 |  | *BARX1* | 1.282051 | 1.730104 |
| *BCORL1* | 6.410256 | 8.304498 |  | *BCL2* | 2.564103 | 2.768166 |  | *THBS1* | 1.282051 | 8.99654 |
| *RBMX* | 6.410256 | 2.076125 |  | *AXIN1* | 2.564103 | 5.882353 |  | *NR2F6* | 1.282051 | 1.384083 |
| *THSD7B* | 6.410256 | 16.609 |  | *PTEN* | 2.564103 | 10.38062 |  | *MEIS1* | 1.282051 | 5.882353 |
| *TET2* | 6.410256 | 4.844291 |  | *ABCA10* | 2.564103 | 9.342561 |  | *TMEM63A* | 1.282051 | 6.920415 |
| *NOTCH1* | 6.410256 | 11.41869 |  | *LARP4B* | 2.564103 | 11.41869 |  | *CNGA4* | 1.282051 | 6.574394 |
| *GNAS* | 6.410256 | 8.99654 |  | *CPD* | 2.564103 | 5.536332 |  | *NR1H4* | 1.282051 | 5.536332 |
| *SPTB* | 6.410256 | 9.342561 |  | *TCF4* | 2.564103 | 7.612457 |  | *HNF1A* | 1.282051 | 4.152249 |
| *RNF43* | 6.410256 | 17.30104 |  | *AKT1* | 2.564103 | 2.076125 |  | *TRIM23* | 1.282051 | 3.806228 |
| ***SETBP1*** | 6.410256 | 13.49481 |  | *SEC63* | 2.564103 | 6.920415 |  | *PDGFRB* | 1.282051 | 5.882353 |
| *DCLK1* | 6.410256 | 15.91696 |  | *IWS1* | 2.564103 | 6.574394 |  | *TXNRD1* | 1.282051 | 3.806228 |
| ***GLI3*** | 5.128205 | 19.72318 |  | *MED12* | 2.564103 | 7.958478 |  | *SMAD2* | 1.282051 | 4.844291 |
| *PLB1* | 5.128205 | 9.342561 |  | *SF3B1* | 2.564103 | 7.266436 |  | *SMARCB1* | 1.282051 | 6.228374 |
| *HDAC4* | 5.128205 | 12.11073 |  | *CYLD* | 2.564103 | 9.688581 |  | *THRAP3* | 1.282051 | 4.844291 |
| *FRMD4A* | 5.128205 | 10.38062 |  | *NCOR1* | 2.564103 | 14.87889 |  | *PBRM1* | 1.282051 | 9.688581 |
| *VPS13A* | 5.128205 | 17.99308 |  | *PPP2R1A* | 2.564103 | 4.844291 |  | *MEN1* | 1.282051 | 3.806228 |
| *NOTCH2* | 5.128205 | 11.76471 |  | *ELF3* | 2.564103 | 4.152249 |  | *DNMT1* | 1.282051 | 9.688581 |
| *PTPRC* | 5.128205 | 11.07266 |  | *MACF1* | 2.564103 | 22.49135 |  | *IL2RG* | 1.282051 | 2.076125 |
| *CTNNB1* | 5.128205 | 10.72664 |  | *CARD11* | 2.564103 | 10.72664 |  | *HLA-A* | 1.282051 | 5.190311 |
| *SOX9* | 5.128205 | 5.190311 |  | *NAA25* | 2.564103 | 10.0346 |  | *JAK1* | 1.282051 | 7.612457 |
| *DNMT3A* | 5.128205 | 5.536332 |  | *VHL* | 2.564103 | 1.384083 |  | *MYCN* | 1.282051 | 1.038062 |
| *CIC* | 5.128205 | 10.38062 |  | *TSC1* | 2.564103 | 7.266436 |  | *PAX6* | 1.282051 | 7.266436 |
| *STAG2* | 5.128205 | 8.304498 |  | *SETD2* | 2.564103 | 8.650519 |  | *CDK6* | 1.282051 | 3.460208 |
| *CTNND1* | 5.128205 | 11.41869 |  | *CUL3* | 2.564103 | 5.882353 |  | *RBM28* | 1.282051 | 5.536332 |
| *TNPO1* | 3.846154 | 5.536332 |  | *KIF13A* | 2.564103 | 8.99654 |  | *CSF1R* | 1.282051 | 7.612457 |
| *BNC2* | 3.846154 | 10.38062 |  | *KIT* | 2.564103 | 5.190311 |  | *PRRX1* | 1.282051 | 3.460208 |
| *ASXL1* | 3.846154 | 8.99654 |  | *MET* | 2.564103 | 8.650519 |  | *PCBP1* | 1.282051 | 1.038062 |
| *KMT2B* | 3.846154 | 14.18685 |  | *SMARCA4* | 2.564103 | 10.38062 |  | *PTPN11* | 1.282051 | 5.190311 |
| *MSH6* | 3.846154 | 8.650519 |  | *ALPK2* | 2.564103 | 8.99654 |  | *NRAS* | 1.282051 | 2.076125 |
| *JAK3* | 3.846154 | 5.882353 |  | *ERG* | 2.564103 | 7.266436 |  | *MAP2K1* | 1.282051 | 3.460208 |
| *KDM6A* | 3.846154 | 9.342561 |  | *CDKN2A* | 2.564103 | 5.536332 |  | *PBX1* | 1.282051 | 8.650519 |
| *ARID1B* | 3.846154 | 12.45675 |  | *ATRX* | 2.564103 | 13.14879 |  | *IDH1* | 1.282051 | 4.152249 |
| *TGFBR2* | 3.846154 | 6.574394 |  | *CASP8* | 2.564103 | 8.99654 |  | *EYA4* | 1.282051 | 8.650519 |
| *CHRD* | 3.846154 | 8.304498 |  | *GRHL2* | 2.564103 | 5.882353 |  | *ARFGAP2* | 1.282051 | 3.114187 |
| *SMAD4* | 3.846154 | 8.99654 |  | *MAP2K4* | 2.564103 | 6.574394 |  | *STAT3* | 1.282051 | 4.844291 |
| *CTNNA2* | 3.846154 | 14.87889 |  | *FLI1* | 2.564103 | 2.768166 |  | *TGIF1* | 1.282051 | 2.076125 |
| *ARID2* | 3.846154 | 10.72664 |  | *HMBOX1* | 1.282051 | 3.114187 |  | *PPARA* | 1.282051 | 2.768166 |
| *EP300* | 3.846154 | 11.07266 |  | *FGFR3* | 1.282051 | 4.152249 |  | *NCOA3* | 1.282051 | 11.07266 |
| *NDRG1* | 3.846154 | 3.806228 |  | *EGFR* | 1.282051 | 11.07266 |  | *CUL1* | 1.282051 | 8.99654 |

Genes were ranked according to their mutation frequencies in MS data.

**Table S2.** The correlation between double-stratifications (WE, RE, WR) of m6A signatures and clinical indexes in MS cohort.

| MS | W^L^E^H^ | W^H^E^L^ | *p* | R^L^E^H^ | R^H^E^L^ | *p* | W^L^R^L^ | W^H^R^H^ | *p* |
| --- | --- | --- | --- | --- | --- | --- | --- | --- | --- |
| Total | 21 | 21 |  | 18 | 18 |  | 24 | 24 |  |
| Gender |  |  | 1.0000 |  |  | 1.0000 |  |  | 1.0000 |
| male | 13 (62) | 12 (57) |  | 12 (67) | 11 (61) |  | 16 (67) | 16 (67) |  |
| female | 8 (38) | 9 (43) |  | 6 (33) | 7 (39) |  | 8 (33) | 8 (33) |  |
| Age |  |  | 1.0000 |  |  | 0.4998 |  |  | 0.5639 |
| <=60 | 12 (57) | 11 (52) |  | 12 (67) | 9 (50) |  | 14 (58) | 11 (46) |  |
| >60 | 9 (43) | 10 (48) |  | 6 (33) | 9 (50) |  | 10 (42) | 13 (54) |  |
| T |  |  | 0.3533 |  |  | 0.1931 |  |  | 0.1132 |
| t1 | 0 (0) | 0 (0) |  | 0 (0) | 0 (0) |  | 0 (0) | 0 (0) |  |
| t2 | 2 (9) | 3 (14) |  | 0 (0) | 3 (17) |  | 0 (0) | 3 (13) |  |
| t3 | 9 (43) | 11 (52) |  | 8 (44) | 7 (39) |  | 11 (46) | 13 (54) |  |
| t4 | 10 (48) | 7 (34) |  | 10 (46) | 8 (44) |  | 13 (54) | 8 (33) |  |
| N |  |  | 0.3118 |  |  | 0.1299 |  |  | 0.6274 |
| n0 | 4 (19) | 6 (28) |  | 2 (11) | 5 (28) |  | 5 (21) | 6 (25) |  |
| n1 | 4 (19) | 5 (24) |  | 3 (17) | 4 (22) |  | 5 (21) | 5 (21) |  |
| n2 | 3 (14) | 3 (14) |  | 2 (11) | 2 (11) |  | 4 (16) | 5 (21) |  |
| n3 | 10 (48) | 7 (34) |  | 11 (61) | 7 (39) |  | 10 (42) | 8 (33) |  |
| M |  |  | 1.0000 |  |  | 1.0000 |  |  | 1.0000 |
| m0 | 21 (100) | 21 (100) |  | 18 (100) | 18 (100) |  | 23 (96) | 24 (100) |  |
| m1 | 0 (0) | 0 (0) |  | 0 (0) | 0 (0) |  | 1 (4) | 0 (0) |  |
| Stage |  |  | 0.2401 |  |  | 0.0580 |  |  | 0.0250 |
| I | 1 (5) | 2 (9) |  | 0 (0) | 2 (11) |  | 0 (0) | 2 (8) |  |
| II | 5 (24) | 7 (34) |  | 3 (17) | 5 (28) |  | 5 (21) | 8 (34) |  |
| III | 14 (66) | 12 (57) |  | 14 (78) | 11 (61) |  | 16 (67) | 14 (58) |  |
| IV | 1 (5) | 0 (0) |  | 1 (5) | 0 (0) |  | 3 (12) | 0 (0) |  |

High m6A-indications (W^H^E^L^, R^H^E^L^, W^H^R^H^) were compared with low m6A-indications (W^L^E^H^, R^L^E^H^, W^L^R^L^). Statistics were performed with Fisher’s exact test or Chi-square test. **Table S3.** The correlation between double-stratifications (WE, RE, WR) of m6A signatures and clinical indexes in TCGA cohort.

| TCGA | W^L^E^H^ | W^H^E^L^ | *p* | R^L^E^H^ | R^H^E^L^ | *p* | W^L^R^L^ | W^H^R^H^ | *p* |
| --- | --- | --- | --- | --- | --- | --- | --- | --- | --- |
| Total | 42 | 43 |  | 60 | 60 |  | 87 | 88 |  |
| Gender |  |  | 0.2701 |  |  | 1.0000 |  |  | 0.7563 |
| male | 28 (67) | 23 (53) |  | 36 (60) | 35 (58) |  | 55 (63) | 53 (60) |  |
| female | 14 (33) | 20 (47) |  | 24 (40) | 25 (42) |  | 32 (37) | 35 (40) |  |
| Age |  |  | 0.1310 |  |  | 0.0067 |  |  | 0.5156 |
| <=60 | 13 (31) | 7 (16) |  | 23 (38) | 9 (15) |  | 29 (33) | 25 (28) |  |
| >60 | 29 (69) | 36 (84) |  | 37 (62) | 51 (85) |  | 58 (67) | 63 (72) |  |
| Lauren |  |  | 0.0009 |  |  | 0.0000 |  |  | 0.0039 |
| diffuse | 15 (36) | 3 (7) |  | 29 (48) | 4 (7) |  | 29 (33) | 13 (15) |  |
| intestinal | 21 (50) | 35 (81) |  | 26 (43) | 54 (90) |  | 48 (55) | 65 (74) |  |
| others | 6 (14) | 5 (12) |  | 5 (9) | 2 (3) |  | 10 (11) | 10 (11) |  |
| T |  |  | 0.0424 |  |  | 0.1371 |  |  | 0.0221 |
| t1 | 0 (0) | 3 (7) |  | 0 (0) | 4 (7) |  | 2 (2) | 8 (9) |  |
| t2 | 5 (12) | 7 (17) |  | 12 (20) | 10 (17) |  | 16 (18) | 13 (15) |  |
| t3 | 29 (69) | 16 (37) |  | 33 (55) | 24 (40) |  | 54 (63) | 38 (43) |  |
| t4 | 8 (19) | 13 (30) |  | 14 (23) | 16 (26) |  | 15 (17) | 26 (30) |  |
| n/a | 0 (0) | 4 (9) |  | 1 (2) | 6 (10) |  | 0 (0) | 3 (3) |  |
| N |  |  | 0.0760 |  |  | 0.0886 |  |  | 0.7442 |
| n0 | 14 (33) | 15 (35) |  | 23 (38) | 16 (26) |  | 29 (33) | 32 (36) |  |
| n1 | 7 (17) | 5 (12) |  | 13 (22) | 9 (15) |  | 22 (25) | 18 (20) |  |
| n2 | 5 (12) | 11 (25) |  | 8 (13) | 18 (30) |  | 16 (18) | 13 (15) |  |
| n3 | 16 (38) | 6 (14) |  | 15 (25) | 10 (17) |  | 19 (23) | 19 (22) |  |
| n/a | 0 (0) | 6 (14) |  | 1 (2) | 7 (12) |  | 1 (1) | 6 (7) |  |
| M |  |  | 1.0000 |  |  | 1.0000 |  |  | 0.5905 |
| m0 | 39 (93) | 38 (88) |  | 57 (95) | 56 (94) |  | 81 (93) | 78 (89) |  |
| m1 | 3 97) | 3 (7) |  | 3 (5) | 2 (3) |  | 6 (7) | 8 (9) |  |
| n/a | 0 (0) | 2 (5) |  | 0 (0) | 2 (3) |  | 0 (0) | 2 (2) |  |
| Stage |  |  | 0.4290 |  |  | 0.5018 |  |  | 0.1159 |
| I | 4 (10) | 6 (14) |  | 6 (10) | 8 (13) |  | 9 (10) | 17 (19) |  |
| II | 14 (33) | 12 (28) |  | 29 (48) | 18 (30) |  | 41 (47) | 26 (30) |  |
| III | 21 (50) | 14 (32) |  | 21 (35) | 22 (37) |  | 30 (35) | 29 (33) |  |
| IV | 3 (7) | 3 (7) |  | 3 (5) | 2 (3) |  | 6 (7) | 8 (9) |  |
| n/a | 0 (0) | 8 (19) |  | 1 (2) | 10 (17) |  | 1 (1) | 8 (9) |  |

High m6A-indications (W^H^E^L^, R^H^E^L^, W^H^R^H^) were compared with low m6A-indications (W^L^E^H^, R^L^E^H^, W^L^R^L^). Statistics were performed with Fisher’s exact test or Chi-square test.

**Table S4.** The correlation between triple-stratification (WRE) of m6A signatures and clinical indexes for diverse Lauren subtypes in TCGA cohort.

|  | diffuse | |  | intestinal | |  | others | |  |
| --- | --- | --- | --- | --- | --- | --- | --- | --- | --- |
| TCGA | WR^dL^E^H^ | WR^dH^E^L^ | *p* | WR^dL^E^H^ | WR^dH^E^L^ | *p* | WR^dL^E^H^ | WR^dH^E^L^ | *p* |
| Total | 13 | 2 |  | 13 | 26 |  | 3 | 2 |  |
| Gender |  |  | 0.4667 |  |  | 1.0000 |  |  | 0.4000 |
| male | 7 (54) | 0 (0) |  | 9 (69) | 17 (65) |  | 2 (67) | 0 (0) |  |
| female | 6 (46) | 2 (100) |  | 4 (31) | 9 (35) |  | 1 (33) | 2 (100) |  |
| Age |  |  | 0.1429 |  |  | 1.0000 |  |  | - |
| <=60 | 9 (69) | 0 (0) |  | 2 (15) | 4 (15) |  | 0 (0) | 0 (0) |  |
| >60 | 4 (31) | 2 (100) |  | 11 (85) | 22 (85) |  | 3 (100) | 2 (100) |  |
| Lauren |  |  | - |  |  | - |  |  | - |
| diffuse | 13 (100) | 2 (100) |  | 0 (0) | 0 (0) |  | 0 (0) | 0 (0) |  |
| intestinal | 0 (0) | 0 (0) |  | 13 (100) | 26 (100) |  | 0 (0) | 0 (0) |  |
| others | 0 (0) | 0 (0) |  | 0 (0) | 0 (0) |  | 3 (100) | 2 (100) |  |
| T |  |  | 0.4308 |  |  | 0.0484 |  |  | 0.8333 |
| t1 | 0 (0) | 0 (0) |  | 0 (0) | 3 (11) |  | 0 (0) | 0 (0) |  |
| t2 | 0 (0) | 0 (0) |  | 4 (31) | 6 (23) |  | 0 (0) | 0 (0) |  |
| t3 | 9 (69) | 1 (50) |  | 9 (69) | 8 (31) |  | 1 (33) | 0 (0) |  |
| t4 | 4 (31) | 0 (0) |  | 0 (0) | 7 (27) |  | 2 (67) | 2 (100) |  |
| n/a | 0 (0) | 1 (50) |  | 0 (0) | 2 (8) |  | 0 (0) | 0 (0) |  |
| N |  |  | 0.3433 |  |  | 0.2188 |  |  | - |
| n0 | 3 (23) | 0 (0) |  | 7 (54) | 9 (35) |  | 0 (0) | 0 (0) |  |
| n1 | 0 (0) | 0 (0) |  | 4 (31) | 4 (15) |  | 0 (0) | 0 (0) |  |
| n2 | 4 (31) | 1 (50) |  | 0 (0) | 6 (23) |  | 0 (0) | 0 (0) |  |
| n3 | 6 (46) | 1 (50) |  | 2 (15) | 3 (12) |  | 3 (100) | 1 (50) |  |
| n/a | 0 (0) | 0 (0) |  | 0 (0) | 4 (15) |  | 0 (0) | 1 (50) |  |
| M |  |  | 0.3714 |  |  | 1.0000 |  |  | - |
| m0 | 11 (85) | 1 (50) |  | 13 (100) | 24 (92) |  | 3 (100) | 1 (50) |  |
| m1 | 2 (15) | 1 (50) |  | 0 (0) | 1 (4) |  | 0 (0) | 0 (0) |  |
| n/a | 0 (0) | 0 (0) |  | 0 (0) | 1 (4) |  | 0 (0) | 1 (50) |  |
| Stage |  |  | 0.1129 |  |  | 0.2369 |  |  | - |
| I | 0 (0) | 0 (0) |  | 3 (23) | 6 (23) |  | 0 (0) | 0 (0) |  |
| II | 3 (23) | 0 (0) |  | 8 (62) | 6 (23) |  | 0 (0) | 0 (0) |  |
| III | 8 (62) | 0 (0) |  | 2 (15) | 8 (30) |  | 3 (100) | 1 (50) |  |
| IV | 2 (15) | 1 (50) |  | 0 (0) | 1 (4) |  | 0 (0) | 0 (0) |  |
| n/a | 0 (0) | 1 (50) |  | 0 (0) | 5 (20) |  | 0 (0) | 1 (50) |  |

For patients with different Lauren subtypes (diffuse, intestinal, others), high m6A-indication (WR^dH^E^L^) was compared with low m6A-indication (WR^dL^E^H^). Statistics were performed with Fisher’s exact test or Chi-square test.
